# A progressive three-state model to estimate time to cancer: a likelihood-based approach

**DOI:** 10.1186/s12874-022-01645-2

**Published:** 2022-06-27

**Authors:** Eddymurphy U. Akwiwu, Thomas Klausch, Henriette C. Jodal, Beatriz Carvalho, Magnus Løberg, Mette Kalager, Johannes Berkhof, Veerle M. H. Coupé

**Affiliations:** 1 Amsterdam UMC, Vrije Universiteit Amsterdam, Department of Epidemiology and Data Science, Amsterdam Public Health, Amsterdam, The Netherlands; 2 Clinical Effectiveness Research Group, University of Oslo and Oslo University Hospital, Oslo, Norway; 3 Department of Pathology, Netherlands Cancer Institute, Amsterdam, The Netherlands

**Keywords:** Colorectal cancer, Colorectal cancer surveillance, Adenoma, Adenoma surveillance, Progressive three-state disease model, Simulation, Maximum likelihood, Adenoma-carcinoma sequence, Interval-censored data

## Abstract

**Background:**

To optimize colorectal cancer (CRC) screening and surveillance, information regarding the time-dependent risk of advanced adenomas (AA) to develop into CRC is crucial. However, since AA are removed after diagnosis, the time from AA to CRC cannot be observed in an ethically acceptable manner. We propose a statistical method to indirectly infer this time in a progressive three-state disease model using surveillance data.

**Methods:**

Sixteen models were specified, with and without covariates. Parameters of the parametric time-to-event distributions from the adenoma-free state (AF) to AA and from AA to CRC were estimated simultaneously, by maximizing the likelihood function. Model performance was assessed via simulation. The methodology was applied to a random sample of 878 individuals from a Norwegian adenoma cohort.

**Results:**

Estimates of the parameters of the time distributions are consistent and the 95% confidence intervals (CIs) have good coverage. For the Norwegian sample (AF: 78*%*, AA: 20*%*, CRC: 2*%*), a Weibull model for both transition times was selected as the final model based on information criteria. The mean time among those who have made the transition to CRC since AA onset within 50 years was estimated to be 4.80 years (95% CI: 0; 7.61). The 5-year and 10-year cumulative incidence of CRC from AA was 13.8*%* (95*%* CI: 7.8*%*;23.8*%*) and 15.4*%* (95*%* CI: 8.2*%*;34.0*%*), respectively.

**Conclusions:**

The time-dependent risk from AA to CRC is crucial to explain differences in the outcomes of microsimulation models used for the optimization of CRC prevention. Our method allows for improving models by the inclusion of data-driven time distributions.

**Supplementary Information:**

The online version contains supplementary material available at (10.1186/s12874-022-01645-2).

## Background

With over 1.9 million new cases of colorectal cancer (CRC) in 2020, CRC is the second and third most common cancer worldwide in women and men, respectively [1]. CRC mortality has been declining for a number of years, possibly due to improved surgery, the administration of adjuvant therapy, and earlier diagnosis. Unarguably, one of the best ways to reduce CRC death is by early detection of both adenomatous polyps and early-stage cancer [2, 3]. Screening asymptomatic individuals with the removal of all detected adenomas, followed by post-polypectomy surveillance, has been shown to be effective in reducing CRC incidence and/or mortality [4–8]. To evaluate the effect of CRC screening and surveillance on long-term CRC mortality, intermediate endpoints are currently used. Advanced adenoma (AA), defined as an adenoma with a villous component, high-grade dysplasia and/or size >=10 mm, is the most used intermediate endpoint [7, 9, 10]. How well the use of an intermediate endpoint leads to sensible conclusions for the effect of screening on cancer incidence and mortality is unclear. Previous research has suggested that AA is a more valid surrogate marker for CRC risk than adenomas since AA has a greater potential to develop into cancer [11, 12]. Estimation of the time distribution from AA to CRC is important to predict long-term outcomes of screening and surveillance programs in the absence of observations. It has been shown to be a crucial parameter explaining differences in the outcomes of microsimulation models used to study CRC screening [13]. In addition, information regarding time to CRC has been described as a key consideration when evaluating surveillance after polypectomy [14]. Specifically, a short average interval from AA to CRC would mean that individuals would need to be screened at shorter rather than longer intervals and vice-versa. However, this time to event is impossible to observe in an ethically acceptable manner and hence estimating its distribution is in all but the simple exponential case [15, 16]. Given that CRC development can be described as a progressive disease process from a healthy adenoma-free (AF) state to AA and finally CRC, the concept of multi-state statistical modelling is relevant.

In the past, different multistate models with varying assumptions have been proposed to study disease natural history. These models differ in terms of the type of disease process, data structure, observation process, probability distributions used, and estimation (see Methods section). Motivated by CRC surveillance, this paper considers the particular problem of indirectly inferring the time from AA to CRC in a progressive three-state disease model where an individual is censored once the second health state is observed to occur. Although this means that the second transition time is never directly observed, both transition times can be jointly estimated. For this, we derive and maximize the joint likelihood function, in which the two transition times could assume any probability distribution without covariates, any of the three parametric proportional hazards (PH) models (i.e., exponential, Weibull and Gompertz [17]) if covariate-dependent, and be either left-, right- or interval-censored. We illustrate our method by specifying 16 different models, with and without covariates, assuming an exponential or Weibull distribution. Furthermore, we demonstrate that the censored time from AA to CRC can be correctly estimated using simulated and real data examples. In the Additional file 1, we provide the **R** code with a full description of how to implement our method.

## Methods

### Related multi-state models

Several multi-state models have been proposed to describe disease processes with different assumptions on (1) the type of data structure, (2) the observation process, and (3) the methodological approach in estimating the time distributions between health states [18–33].

With respect to the type of data structure,[19, 30, 32, 33] two methods proposed by Vink et al. [19] in human papillomavirus (HPV) screening and Yen et al. [30] using a CRC frailty model, are based on current status data where the health state of individuals is only observed at a single examination, making it impossible to observe the disease process over time. The type of data structure from the Norwegian adenoma cohort [34, 35], motivating our method, is based on CRC surveillance where individuals are periodically examined over time, leading to interval-censored data with intervals of varying lengths (also called *panel* data).

In terms of the observation process, several methods proposed by, for example, Kapetanakis et al. [24]; Titman and Sharples [25]; Van Den Hout [28]; and Joly and Commenges [26], assume three-state semi-Markov models. Contrary to our interval-censored setting, these models assume that in the presence of interval-censored transitions from state 1 to state 2, the exact time to state 3 is observed. Foucher et al. [29] postulated a similar observation process but used more than three health states, again with the entry to the final state being exactly known. A more general observation process resulting from the so-called *doubly censored* data in a three-state model was introduced by De Gruttola and Lagakos [20]; Gómez and Lagakos [22]; and Kim et al. [21] for studying HIV/AIDS. In these studies, the time intervals at which states 2 and 3 occur are both observed separately in the same individual. These models can be implemented using the **p3state.msm R** package [36]. A very specific case of doubly censored data was studied by Griffin and Lagakos [31], where the length of the intervals between two consecutive visits must not vary across individuals and state 2 may be observed multiple times until the individual is censored either by reaching state 3 or through right-censoring at the last visit. None of the above methods address the particularity of the data collected during surveillance in some cancer types, such as CRC surveillance. That is, the observation process is not only interval-censored at both transition times (i.e., state 1 → state 2 and state 1 → state 3), but the second transition is never directly observed because individuals are censored (treated) once state 2 is detected.

In terms of methodological approach, existing methods differ with respect to the assumptions made for the sojourn time distributions between health states [18, 23, 25, 27]. For instance, a method proposed by Straatman et al. [18] for fitting breast cancer screening models is limited to exponential distributions for the sojourn times. Similarly, Wei and Kryscio [27] suggested a model where all transition from the baseline state were constrained to be exponential. Jackson et al. [23] developed a more flexible method in the **msm** package in **R**, using a user-defined piecewise-constant hazard model, that allows more general censoring mechanisms in approximating an arbitrary sojourn time distribution from baseline. However, the piecewise-constant hazard model is only applicable to time since the beginning of the process (i.e., baseline) and would not be applicable to time since the previous health state (e.g., state 2 → state 3), like in a semi-Markov model. An alternative method using Coxian phase-type distributions was presented by Titman and Sharples [25]. The two-phase semi-Markov model can be implemented using the phase.states() option to **R msm** package version 1.6.9. In this paper, we compare our method to the method by Titman and Sharples [25] as this is the only available method that can fit a semi-markov model with three health states like ours. Our results indicate that when using a data structure and disease process as ours where state 2 to 3 is never directly observed, the method by Titman and Sharples [25] fits poorly for the second not directly-observable transition time (i.e., state 2 → state 3) when a non-exponential distribution is specified for state 2 to 3.

While the aforementioned literature is rich with methods for estimating transition times in multi-state models, they either provide the user with limited probability distribution options, or do not adequately accommodate the type of data arising from cancer surveillance where the transition from the pre-final to final state is not observed in individuals for whom the pre-final state is detected. The method presented in this paper seeks to address these limitations by providing a more suitable method for estimating the transition times in a three-state model using CRC surveillance data where state 2 to 3 is never observed. Our objective is model the sojourn time distribution by a parametric PH model assuming either exponential, Weibull, or Gompertz distributions, where the “best” model can be chosen using model selection and/or goodness-of-fit criteria [37].

### Notation and assumptions

Let *X* and *Y* be two random variables that may be independent or conditionally independent given covariates ***w***. Variables *X* and *Y* denote the transition times in a progressive three-state model with health states consisting of those individuals that are AF; that is, without AA or CRC; and those with either AA or CRC. These health states are hereafter referred to as states AF, AA, and CRC, respectively. Variables *X* and *Y* denote the durations from AF to AA and from AA to CRC respectively, and their sum, *Z*=*X*+*Y*, denotes the duration from AF to CRC. We denote *f*(*x*) and *f*(*x*|***w***) as the marginal and conditional probability density functions (PDFs) of *X*, respectively; and *g*(*y*) and *g*(*y*|***w***) as the marginal and conditional PDFs of *Y*, respectively. Similarly, let *F*(*x*),*F*(*x*|***w***),*G*(*y*), and *G*(*y*|***w***) denote the corresponding cumulative distribution functions (CDFs). Also, we assume that AAs do not regress and that all CRCs develop from AA. Similar assumptions have implicitly or explicitly been made or suggested by some authors [11, 16], and it seems plausible on biological grounds [38, 39]. We further assume that AFs include non-advanced adenomas (NAAs). After baseline colonoscopy, the first colonoscopy that leads to inclusion into the cohort, all individuals with colorectal polyps of any size are considered successfully treated by means of polypectomy prior to the start of surveillance. As such, everyone starts in the AF state with no CRC at baseline. During surveillance, individuals are followed-up with repeat colonoscopy or sigmoidoscopy, followed by complete colonoscopy in case of positive findings, according to a predefined schedule that may or may not be exactly followed with respect to timing of the visit. This means that the schedule is allowed to vary across individuals. We assume that the surveillance test or combination of tests is perfect, that is, its sensitivity and specificity are 100*%*. Although particularly smaller, flat or sessile lesions may be missed on colonoscopy, this assumption is reasonable for AAs, which are generally ≥ 10 mm in size, and CRC [40, 41]. An individual is either left-censored when detected with either AA or CRC at the first surveillance visit after baseline, right-censored if AF is reported at the end of the follow-up, and interval-censored when AF is followed by AA or CRC at the next visit [42].

### Model

**Fig. 1:**
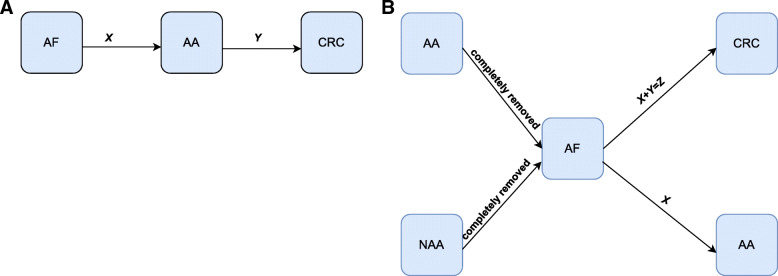
Multi-state model of colorectal cancer. (**A**) Natural history process, (**B**) Observed transition pathways

Based on the above assumptions, we propose the following three-state model with irreversible transitions as shown in Fig. 1. Figure 1a shows the assumed underlying natural history disease process during the surveillance period. In the assumed disease process, which is based on the adenoma-carcinoma sequence [43], individuals progress to CRC through the AA state. However, this underlying process is not observed in reality because during each surveillance interval, individuals may rapidly progress to the CRC state without being detected in the AA state. Moreover, if an individual is being detected in the AA state, such an individual is censored since the AA is treated (i.e., removed) and the pathway to CRC is effectively closed. In other words, for each individual in the surveillance program, we can only obtain the time information as depicted in Fig. 1b, that is, we observe that from the AF state a transition has been made to the AA state or to the CRC state after one or more surveillance rounds. Note that the exact timing of the transitions is unknown but is known to lie within a given interval after one or more surveillance rounds [44, 45]. Nevertheless, we may infer the assumed process from the observed process as follows: by using the patient-time data from AF to AA and from AF to CRC, we can estimate the time distribution from AA to CRC.

**Fig. 2:**
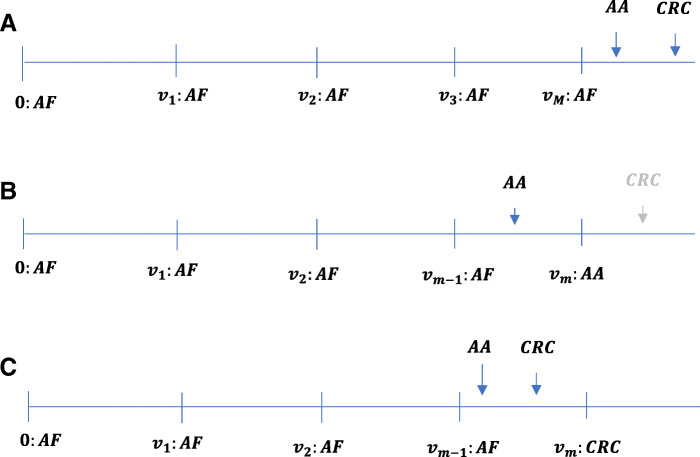
Schematic representation of 3 possible observation process leading to right-censoring (A) and interval- censoring (B and C). From top to bottom, all individuals are AF at time zero prior to start of surveillance (**A**) and remain AF until the end of their follow-up *v*_*m*_, (**B**) detected to be AA, or (**C**) detected to be CRC

There are 3 typical situations that may occur during surveillance which are shown in Fig. 2. Let ***v***=(*v*_1_,*v*_2_,⋯,*v*_*m*−1_,*v*_*m*_) be the vector of ordered visit times that are independent of *X* and *Y* for any given individual. First, AA or CRC is not observed within the period of follow-up, and therefore, if it occurs, this is after the last visit *v*_*m*_. Second, AA is observed within the follow-up period, but CRC is not. Third, CRC is observed within the follow-up period, which necessarily means AA has occurred within the same surveillance interval.

For the situation in Fig. 2a, it can be shown that the chance of an individual not having AA or CRC (i.e., being AF) until and including follow-up visit *v*_*m*_ is given by

1 $$ \Pr \left(X>v_{m} \right) = 1- F(v_{m}).   $$

Similarly, for the situation in Fig. 2b, it can be shown that the chance of an individual having AA but no CRC between two consecutive follow-up visits *v*_*m*−1_ and *v*_*m*_ is given by

2 $$\begin{array}{*{20}l} &\Pr \Big(v_{m-1} <X < v_{m}, Z>v_{m} \Big)  \\ &= F(v_{m}) -F(v_{m-1})-\int_{v_{m-1}}^{v_{m}} f(x) G(v_{m} - x) dx.  \end{array} $$

Lastly, for the situation in Fig. 2c, it can be shown that the chance of an individual having CRC between two consecutive follow-up visits *v*_*m*−1_ and *v*_*m*_ is given by

3 $$\begin{array}{*{20}l} &\Pr \Big(v_{m-1} < X < v_{m}, v_{m-1} <Z < v_{m} \Big)  \\ &=\int_{v_{m-1}}^{v_{m}} f(x) G(v_{m} - x) dx.  \end{array} $$

Derivations of Eqs. (1) to (3) are reported in Section A of the Additional file 2.

### Likelihood construction and estimation

Let $\boldsymbol {v^{(i)}} = \left (v_{1}^{(i)}, v_{2}^{(i)}, v_{3}^{(i)}, \dots, v_{m}^{(i)} \right)$ denote the vector of ordered surveillance visit times for an individual *i* such that

$$ 0 < v_{1}^{(i)} < v_{2}^{(i)} < v_{3}^{(i)} < \cdots < v_{m}^{(i)} < \infty, $$

where *m* is a random number of follow-up observation times for individual *i* after which he/she is censored. We also define the censoring indicators $\boldsymbol {\Delta }_{AA}^{(i)}= 1$ and $\boldsymbol {\Delta }_{CRC}^{(i)}= 1$ if an individual *i* was detected with either AA or CRC, respectively; and 0 otherwise.

The joint log-likelihood for *n* individuals given the observed data $\mathcal {D}^{(i)} = \Big \{ \Bigl (\boldsymbol {w}^{(i)}, v^{(i)}_{m-1}, v^{(i)}_{m}, {\boldsymbol {\Delta }_{AA}^{(i)}},{\boldsymbol {\Delta }_{CRC}^{(i)}} \Bigr); \allowbreak i = 1,2, \cdots, n \Big \} $, expressed as a function of vector of unknown model parameters ***ψ***, is given by

4 $$ \ell (\boldsymbol{\psi}) = \sum\limits_{i=1}^{n} \log \mathcal{L}_{i}(\boldsymbol{\psi}\Big|\mathcal{D}^{(i)}),   $$

where the likelihood contribution of individual *i* with vector of covariates ***w***^(*i*)^ is

5 $$ \begin{aligned} \mathcal{L}_{i}(\boldsymbol{\psi} \left|\mathcal{D}^{(i)})\right. =& \left\{ \Pr \left(v_{m-1}^{(i)} <X < v_{m}^{(i)}, Z>v_{m}^{(i)} \Big| \psi_{x},\psi_{y},\boldsymbol{ w}^{(i)} \right) \right\}^{\boldsymbol{\Delta}_{AA}^{(i)}} \\ & \times \left\{\!\Pr\! \left(v_{m-1}^{(i)} \!<\! X \!<\! v_{m}^{(i)}, \!v_{m-1}^{(i)} \!<Z\! < \!v_{m}^{(i)} \!\left| \!\psi_{x},\psi_{y},\boldsymbol{ w}^{(i)}\! \right) \!\right.\right\}^{\boldsymbol{\Delta}_{CRC}^{(i)}} \\ & \times \left\{ \Pr \left(X >v^{(i)}_{m} \Big| \psi_{x},\psi_{y},\boldsymbol{ w}^{(i)} \right) \right\}^{1-{\boldsymbol{\Delta}_{AA}^{(i)}} -{\boldsymbol{\Delta}_{CRC}^{(i)}}} \\ =& \left\{ \int_{v_{m-1}^{(i)}}^{v_{m}^{(i)}} f(x \left|\psi_{x},\boldsymbol{w}^{(i)}) \left[1-G(v_{m}^{(i)} - x\right|\psi_{y},\boldsymbol{w}^{(i)})\right] dx\right\}^{\boldsymbol{\Delta}_{AA}^{(i)}} \\ & \times \left\{ \int_{v_{m-1}^{(i)}}^{v_{m}^{(i)}} f(x\left|\psi_{x},\boldsymbol{w}^{(i)}) G(v_{m}^{(i)} - x\right|\psi_{y},\boldsymbol{w}^{(i)}) dx \right\}^{\boldsymbol{\Delta}_{CRC}^{(i)}} \\ & \times \left\{ 1- F\left(v^{(i)}_{m}\left|\psi_{x},\boldsymbol{w}^{(i)}\right)\right.\right\}^{1-{\boldsymbol{\Delta}_{AA}^{(i)}} -{\boldsymbol{\Delta}_{CRC}^{(i)}}}.  \end{aligned}  $$

In the above likelihood contributions, $ v^{(i)}_{m-1}$ is the most recent visit time at which an individual *i* is observed to be AF or cancer-free (note that $ v^{(i)}_{m-1} = 0$ if an individual *i* is left-censored), whereas $v^{(i)}_{m} $ is either the time when an individual *i* is detected with an event (i.e., AA or CRC) or the last visit time for a right-censored individual *i*. Also, ***ψ***=*ψ*_*x*_∪*ψ*_*y*_ is defined as the joint set of parameters of the probability distribution of *X* and *Y*, respectively. Eq. 4 is maximized using the optim() function in the statistical software **R**, version 4.0.1 [46], to obtain the maximum likelihood (ML) estimates $\hat {\boldsymbol {\psi }}$ for the parameters ***ψ*** in the model. These estimates are guaranteed to be close to the true ***ψ*** when the sample size *n* is large enough, and the maximization is successful. See Sections B, C and D of the Additional file 2 for inclusion of covariates in the likelihood function, probability expressions in the likelihood function for models without covariates and implementation details, respectively.

### Simulation studies

A series of simulation studies were carried out to investigate the empirical performance of the proposed method and the reliability of the ML estimates obtained. A Monte Carlo (MC) simulation with *N*_*sim*_=1000 runs was carried out for each model specification throughout the study. The study was performed to see whether our proposed method can recover the true parameter values when the true model is fitted to a dataset simulated from that model. We constructed two parameter settings to assess performance under two assumptions for the proportion of CRCs in the cohort (i.e., Scenario I and II), and considered sample sizes of *n*=1000 and 5000. Scenario I was loosely based on Chen et al. [14]: about 55% AFs, 40% AAs and 5% CRCs, and Scenario II: about 30% AFs, 40% AAs and 30% CRCs. A total of 16 different models were specified (Table 1). Model performance was assessed in terms of the root mean squared error (RMSE); relative bias (RB); coefficient of variability (CV), the ratio of empirical standard error (SE) to the true parameter value; empirical coverage rate (CR) of a Wald-based 95% confidence interval (CI), the proportion of the estimated CIs that contain the true parameter value *ψ*; and average CI width (AW) [47–49]. These performance measures are defined as follows

$$\begin{array}{*{20}l} RMSE &= \sqrt{ \frac{1}{N_{sim}} \sum\limits_{j=1}^{N_{sim}} \Big\{ \hat{\psi_{j}} -\psi \Big\}^{2} },\\ RB &= \frac{1}{\psi} \times \left(\frac{1}{N_{sim}} \sum\limits_{j=1}^{N_{sim}} \hat{\psi_{j}} - \psi \right) \times 100,\\ CV &= \frac{1}{\psi} \times \sqrt{\frac{1}{N_{sim}-1} \sum\limits_{j=1}^{N_{sim}} \Big\{ \hat{\psi_{j}} -\bar{\psi} \Big\}^{2} },\\ CR &= \frac{1}{N_{sim}} \sum\limits_{j=1}^{N_{sim}} \Big\{ 1~ \text{if}~ \psi \in \hat{\psi_{j}} \pm 1.96\\ & \times \hat{SE} (\hat{\psi_{j}}), 0 ~\text{otherwise} \Big\},\\ AW &= \frac{1}{N_{sim}} \sum\limits_{j=1}^{N_{sim}} \Big\{2 \times 1.96 \times \hat{SE} (\hat{\psi_{j}}) \Big\}, \end{array} $$

**Table 1:** Percentage of successful simulation runs performed for different model specifications with sample sizes *n*=1000 and 5000, based on 1000 MC simulation runs under Scenarios I and II

					Scenario I		Scenario II	
**Model**	***Pr(x)***	**X** |***w***	***Pr(y)***	**Y** |***w***	***n=1000***	***n=5000***	***n=1000***	***n=5000***
M1	Exponential	No	Exponential	No	100	100	100	100
M2	Exponential	Yes	Exponential	No	100	100	100	100
M3	Exponential	No	Exponential	Yes	100	100	100	100
M4	Exponential	Yes	Exponential	Yes	100	100	100	100
M5	Exponential	No	Weibull	No	100	100	100	100
M6	Exponential	Yes	Weibull	No	100	100	100	100
M7	Exponential	No	Weibull	Yes	100	100	100	100
M8	Exponential	Yes	Weibull	Yes	98.2	91.3	98.2	90.5
M9	Weibull	No	Exponential	No	100	100	100	100
M10	Weibull	Yes	Exponential	No	100	99.6	93.7	90.3
M11	Weibull	No	Exponential	Yes	100	99.9	69.2	71.8
M12	Weibull	Yes	Exponential	Yes	93.8	96.3	79.4	58.1
M13	Weibull	No	Weibull	No	100	100	100	100
M14	Weibull	Yes	Weibull	No	95.2	92.1	99.6	99.5
M15	Weibull	No	Weibull	Yes	100	100	40	47.9
M16	Weibull	Yes	Weibull	Yes	50.8	31.7	68.2	69.5

Pr(*x*): assumed probability distribution of X; Pr(*y*): assumed probability distribution of Y; X |*w*: *X* conditioned on covariates *w*; Y |*w*: Y conditioned on covariates *w*

where $\hat {SE} (\hat {\psi _{j}})$ is the SE of the parameter *ψ* within each simulation run.

To further demonstrate the reliability of the proposed method in fitting semi-Markov models, particularly for the second not directly-observable transition time from state 2 to 3, we performed an additional simulation study where we compared our method to the two-phase semi-Markov model by Titman and Sharples [25] implemented in the **msm R** package version 1.6.9 [23]. We fitted both models using datasets generated under a Weibull probability distribution assumption for both *X* and *Y* without a covariate. Specifically, we used the same parameter settings for model M13 under Scenarios I and II when *n*=5000 in Table 1. Since estimating the second transition time *Y* is our main objective in this paper, more emphasis will be placed on results for *Y* rather than *X*. All simulations were done in the statistical software **R**, version 4.0.1 [46]. See **R** implementation code in the Additional file 1.

#### Data simulation procedure

We created a hypothetical cohort for *i*=1,2,⋯,*n* number of individuals who enter the surveillance after complete removal of their adenomas via colonoscopic polypectomy with *w*^(*i*)^∼*N*(0,1) as covariate. For a chosen model specification for *X* and *Y*, we generated transition times *X* and *Y* for *i*=1,2,⋯,*n* individuals and summed *X* and *Y* to obtain *Z* for each individual *i*. For simplicity, we assumed that the maximum number of endoscopic surveillance visits an individual could have was 4, with such visits generated independently of *X* and *Y* from a uniform distribution over [*a*,*b*], where *a* and *b* are the minimum and maximum years of follow-up of the entire cohort, respectively. For each individual *i*, we compared the observed times *X*^(*i*)^ and *Z*^(*i*)^ previously generated with the individual’s vector of follow-up visit times ***v***^***(i)***^ to obtain the time interval (*v*_*m*−1_,*v*_*m*_] in which AA or CRC must have occurred, and *v*_*m*_ (here, *m*=4) for individuals who had no AA or CRC throughout their follow-up period. For detailed simulation procedure, see Section E of the Additional file 2.

### Data

#### Data structure

Table 2 shows an example dataset, depicting the outcomes of a cohort of individuals based on the steps mentioned above. In this example data set, individuals 2 and 5 were found to have AA during their second and third surveillance visit respectively, while individuals 3,4 and 6 were found to have CRC during their first, second and last surveillance visit, respectively. Finally, individuals may remain free of AA or CRC (i.e, AF state) during their entire follow-up period, as exemplified by individual 1. Hence, Individual 3 is said to be left-censored; Individuals 2,4,5 and 6 interval-censored; while Individual 1 is right-censored.

**Table 2:** Different scenarios of health status of individuals during four follow-up visits after baseline

	Follow-up visits			
Individual	***v***_***1***_	***v***_***2***_	***v***_***3***_	***v***_***4***_
1	AF	AF	AF	AF
2	AF	AA	-	-
3	CRC	-	-	-
4	AF	CRC	-	-
5	AF	AF	AA	-
6	AF	AF	AF	CRC
·	·	·	·	·
·	·	·	·	·
·	·	·	·	·

AF: adenoma-free; AA: advanced adenoma; CRC: colorectal cancer

#### Norwegian adenoma surveillance cohort

**Fig. 3:**
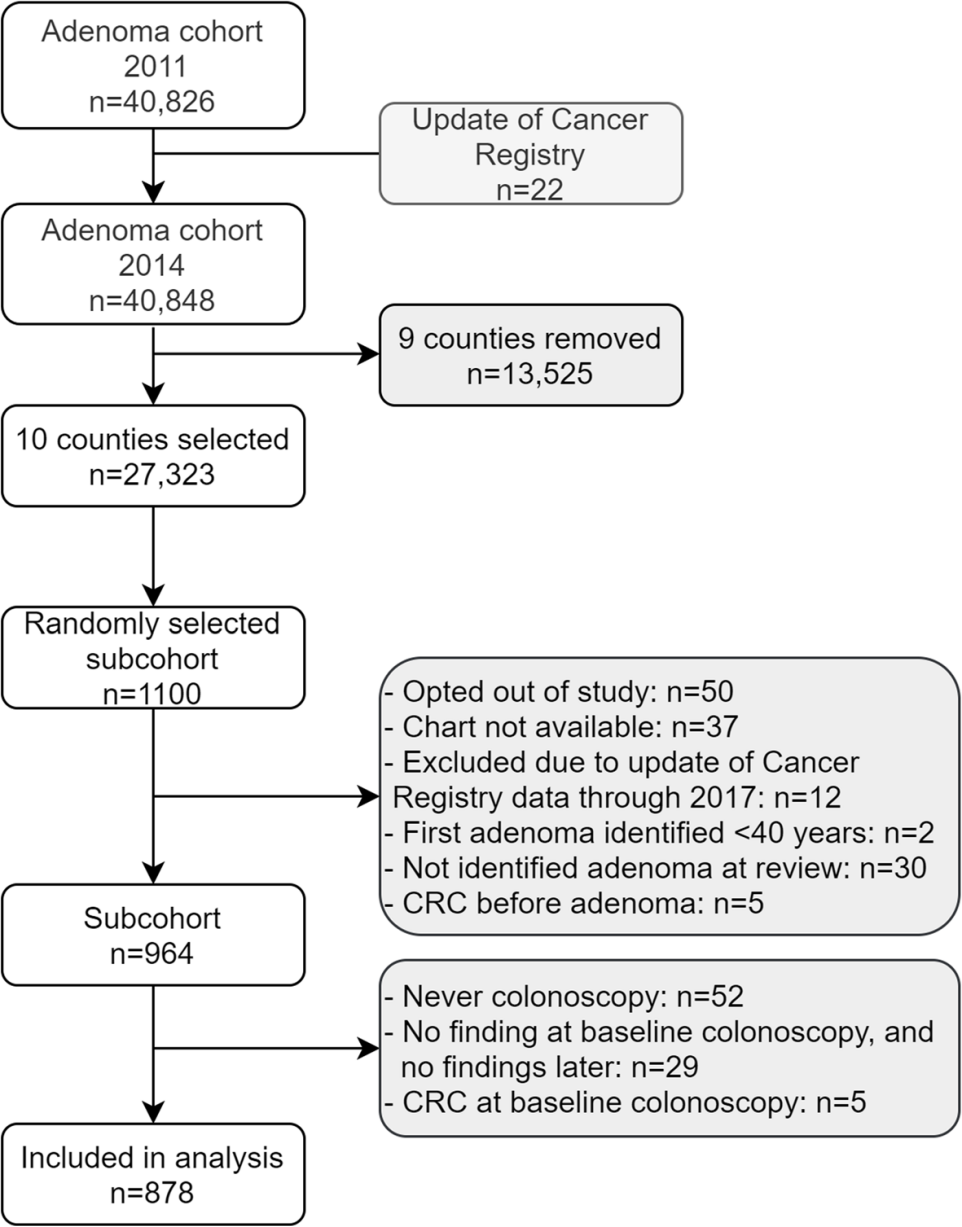
Flow chart of inclusion and exclusion criteria from the adenoma cohort [34, 35]. Never colonoscopy: single entry with non-colonoscopic polypectomy at baseline, or because there was no colonoscopic examination in all visits including baseline. No findings at baseline colonoscopy, and no findings later: a single (i.e., baseline) entry as no finding, or all entries as no findings. CRC at baseline colonoscopy: a single (i.e., baseline) entry as CRC

The adenoma cohort consists of all Norwegian individuals aged 40 years or older, with no previous CRC, who have had adenomas removed between 1993 and 2007 [34, 35]. The entire cohort consists of 40 848 individuals, of whom 1100 individuals were randomly selected for chart review (Fig. 3). The individuals selected for the subcohort were given the opportunity to opt out of the study. Individuals were excluded if they opted out of the study, their chart was not available, the registration in the Cancer Registry was removed at a later update, first adenoma identified <40 years at chart review, they did not have adenomas at chart review, or had CRC preceding their first adenoma. Thus, the subcohort consisted of 964 individuals. For the purpose of this study, each individual’s first colonoscopy was considered the baseline examination, and other endoscopies occurring before this were disregarded. Thus, we excluded any individual who never had a colonoscopy, any individual with no finding at the baseline colonoscopy nor at later endoscopies, and any individual who had CRC at baseline colonoscopy. In total, 878 individuals were included in the data analysis. The retrieved information included dates of follow-up endoscopies with finding (AF, AA or CRC) until 31st December 2017 and patient characteristics such as sex; birth year; adenoma-type (AT) at baseline, i.e., NAA or AA; family history (1st degree relative with CRC); and type of endoscopy used during surveillance. Entry age, family history, sex and AT were included as covariates in the final data analysis. Of the 878 individuals, 688(78.4*%*) were AF until the end of surveillance period, 170(19.4*%*) had AA, and 20(2.2*%*) had CRC during their follow-up. Table 3 shows the distribution of the number of visits for the 878 individuals.

**Table 3:** Distribution of the total number of visits after baseline colonoscopy examination for the 878 individuals in the Norwegian adenoma cohort

**Number of individuals**	205	204	157	115	69	45	26	24	6	9	3	3	6	1	1	1	1	1	1
**Number of visits**	1	2	3	4	5	6	7	8	9	10	11	12	13	15	17	19	23	27	31

## Results

### Simulation results

We first established the number of successful simulation runs for each combination of model specification, sample size *n* and scenario, using MC simulation with *Nsim*=1000 runs (Table 1). Subsequently, the performance of the proposed method was studied in detail. Statistic $\boldsymbol {\hat {\psi }}$ and the corresponding $\hat {SE} (\hat {\psi })$, were calculated in each simulation run per model. The percentage of successful simulation runs is presented in Table 1.

Model complexity and numerical optimization problems, described in section D of the Additional file 2, were reasons for not achieving 100*%* convergence for some models. Resolving these issues requires either changing the starting values or changing the parameters of the optimization algorithm in optim(). Similar issues regarding the success rate of convergence while fitting a model during a MC simulation have been reported elsewhere [50].

As Table 1 shows, the first 15 models under Scenario I resulted in at least 90*%* successful simulation runs. In the remainder, we therefore present detailed results from the simulation studies for these 15 models (Table 4). We observe that for models with exponentially distributed *Y* and no covariates, the absolute values of the RB were less than 1% for all *n*. However, for a higher number of parameters for *Y*, either as a result of being covariate-dependent or because of the use of a Weibull distribution, the absolute values of the RB increased due to low proportion of CRCs. Nevertheless, the absolute values of the RB decreased as the sample size *n* increased across all models. For all models except for model M13, the estimated empirical CRs were approximately close to the 95% nominal coverage when *n* was very large (i.e., *n*=5000) with CRs between 0.936 and 0.964 [47, 49]. The variability of the parameter estimates of *Y* can be examined by means of CV and AW (Table 4). For each model, the absolute values of CV and the AW values of the parameter estimates of *X* were always less than or equal to those of *Y*, and the values of CV and AW decreased as the sample size *n* increased. Finally, the overall accuracy of the estimator can be measured via the RMSE since it incorporates both the bias and variability of the estimator. As the sample size increased, the RMSE value decreased. Results of the estimates of the second transition time *Y* improved further if we changed the simulation setting to include a higher proportion of CRCs, i.e., 30*%* CRC under Scenario II. We report results of models under Scenario II with at least 90*%* successful simulation runs in Supplementary Table S1 in the Additional file 2. Clearly, RB and CV were lower in this setting because of the higher percentage of CRC observed. In summary, the proposed models provided consistent parameter estimates as the RMSE, AW, absolute value of RB and CV decreased as *n* increased. Smaller AWs implied greater accuracy and higher power [48].

**Table 4:** Summary of performance measures for different model specifications under Scenario I, based on 1000 MC simulation runs with sample sizes *n*= 1000 and 5000

			***n=1000***						***n=5000***					
Model	PAR	True	MCM	RMSE	RB^a^	CV	CR	AW	MCM	RMSE	RB^a^	CV	CR	AW
M1	*λ*_1_	0.03	0.03	0.002	0.3	0.05	0.955	0.01	0.03	0.001	0.0	0.01	0.958	0.00
	*λ*_2_	0.04	0.04	0.005	0.1	0.14	0.960	0.02	0.04	0.003	0.0	0.06	0.955	0.01
M2	*α*_0_	− 3.60	− 3.60	0.062	0.0	− 0.02	0.951	0.24	− 3.60	0.027	0.0	−0.01	0.951	0.11
	*α*_1_	− 1.00	−1.00	0.059	0.2	− 0.06	0.942	0.23	− 1.00	0.026	0.2	− 0.03	0.944	0.10
	*λ*_2_	0.04	0.04	0.006	− 0.1	0.15	0.944	0.02	0.04	0.003	0.3	0.07	0.949	0.01
M3	*λ*_1_	0.04	0.03	0.002	− 0.1	0.05	0.955	0.01	0.04	0.001	0.0	0.02	0.946	0.00
	*β*_0_	− 3.20	− 3.22	0.142	0.7	− 0.04	0.967	0.57	− 3.20	0.063	0.2	−0.02	0.952	0.25
	*β*_1_	− 0.10	−0.10	0.152	− 1.9	− 1.52	0.946	0.56	− 0.10	0.062	2.5	− 0.62	0.952	0.25
M4	*α*_0_	− 3.50	− 3.50	0.056	0.0	− 0.02	0.944	0.22	− 3.50	0.025	0.0	−0.01	0.944	0.10
	*α*_1_	− 0.80	−0.80	0.056	0.2	− 0.07	0.944	0.21	− 0.80	0.024	0.2	− 0.03	0.953	0.10
	*β*_0_	− 4.50	− 4.58	0.394	1.7	− 0.09	0.949	1.45	− 4.51	0.155	0.1	−0.03	0.956	0.63
	*β*_1_	− 1.50	−1.56	0.298	3.7	− 0.20	0.948	1.13	− 1.50	0.123	0.2	− 0.08	0.957	0.49
M5	*λ*_1_	0.03	0.03	0.001	0.1	0.05	0.958	0.01	0.03	0.001	0.1	0.01	0.946	0.00
	*κ*_2_	2.00	2.08	0.486	4.0	0.24	0.963	1.82	2.00	0.195	0.2	0.13	0.946	0.77
	*θ*_2_	10.00	10.51	2.966	5.1	0.29	0.928	8.41	10.10	0.768	1.0	0.12	0.959	3.00
M6	*α*_0_	− 4.00	− 4.00	0.084	0.1	− 0.04	0.951	0.34	− 4.00	0.038	0.0	−0.01	0.954	0.15
	*α*_1_	− 2.00	−2.01	0.084	0.3	− 0.03	0.950	0.33	− 2.00	0.038	0.1	− 0.01	0.944	0.15
	*κ*_2_	4.00	4.29	3.327	7.3	0.77	0.959	3.82	4.03	0.355	0.8	0.11	0.956	1.40
	*θ*_2_	8.50	8.56	0.630	0.7	0.14	0.949	2.35	8.51	0.260	0.2	0.04	0.954	1.01
M7	*λ*_1_	0.04	0.04	0.002	− 0.2	0.04	0.950	0.01	0.04	0.001	0.0	0.01	0.950	0.00
	*κ*_2_	4.10	5.67	6.456	38.3	1.51	0.928	9.21	4.25	0.612	3.5	0.11	0.963	2.26
	*β*_0_	2.50	2.51	0.177	0.5	0.13	0.933	0.68	2.50	0.075	− 0.1	0.04	0.936	0.29
	*β*_1_	3.50	4.75	5.065	35.7	1.38	0.933	6.70	3.59	0.437	2.7	0.13	0.963	1.63
M8	*α*_0_	− 3.50	− 3.50	0.060	0.1	− 0.02	0.945	0.23	− 3.50	0.026	0.0	−0.01	0.958	0.10
	*α*_1_	− 1.00	−1.00	0.058	0.2	− 0.06	0.952	0.22	− 1.00	0.026	0.2	− 0.03	0.958	0.10
	*κ*_2_	1.80	1.84	0.408	2.1	0.23	0.955	1.52	1.81	0.171	0.7	0.09	0.945	0.66
	*β*_0_	2.50	2.54	0.227	1.7	0.09	0.946	0.86	2.51	0.090	0.2	0.04	0.954	0.35
	*β*_1_	0.10	0.10	0.184	2.9	1.84	0.956	0.72	0.11	0.082	− 0.5	0.81	0.954	0.31
M9	*κ*_1_	0.40	0.40	0.029	0.2	0.07	0.954	0.11	0.40	0.013	0.0	0.03	0.950	0.05
	*θ*_1_	65.00	66.42	11.453	2.2	0.17	0.956	44.21	65.34	4.993	0.5	0.08	0.951	19.09
	*λ*_2_	0.04	0.04	0.005	0.6	0.14	0.948	0.02	0.04	0.002	0.3	0.06	0.946	0.01
M10	*κ*_1_	2.00	2.02	0.110	0.8	0.05	0.952	0.41	2.00	0.047	0.2	0.02	0.953	0.18
	*α*_0_	3.50	3.50	0.056	0.0	0.02	0.955	0.23	3.50	0.025	0.0	0.01	0.962	0.10
	*α*_1_	3.50	3.52	0.173	0.7	0.05	0.948	0.65	3.51	0.073	0.2	0.02	0.943	0.29
	*λ*_2_	0.04	0.04	0.006	0.4	0.14	0.953	0.02	0.04	0.003	0.2	0.06	0.947	0.01
M11	*κ*_1_	4.00	4.01	0.197	0.3	0.05	0.942	0.76	4.00	0.088	0.1	0.02	0.954	0.34
	*θ*_1_	20.00	20.00	0.300	0.0	0.02	0.936	1.11	20.00	0.130	0.0	0.01	0.939	0.50
	*β*_0_	− 3.50	− 3.55	0.264	1.5	− 0.07	0.960	1.00	− 3.51	0.115	0.2	−0.03	0.941	0.44
	*β*_1_	− 1.50	−1.54	0.234	2.7	− 0.15	0.957	0.89	− 1.51	0.102	0.4	− 0.07	0.949	0.39
M12	*κ*_1_	0.50	0.50	0.039	0.7	0.08	0.961	0.16	0.50	0.018	0.1	0.04	0.944	0.07
	*α*_0_	5.00	5.00	0.247	0.1	0.05	0.954	0.98	5.00	0.112	0.1	0.02	0.941	0.44
	*α*_1_	− 2.00	−2.01	0.092	0.3	− 0.05	0.957	0.37	− 2.00	0.043	0.0	− 0.02	0.945	0.16
	*β*_0_	− 1.00	− 0.96	0.273	− 3.5	− 0.27	0.949	1.02	− 1.00	0.112	− 0.4	−0.11	0.952	0.44
	*β*_1_	5.50	5.67	0.789	3.1	0.14	0.962	2.92	5.53	0.314	0.6	0.06	0.954	1.24
M13	*κ*_1_	1.50	1.50	0.073	0.0	0.05	0.952	0.29	1.50	0.032	−0.1	0.02	0.956	0.13
	*θ*_1_	23.00	23.04	0.880	0.2	0.04	0.940	3.32	23.01	0.375	0.0	0.02	0.945	1.47
	*κ*_2_	0.80	0.81	0.260	0.9	0.32	0.959	1.01	0.81	0.117	0.8	0.15	0.952	0.45
	*θ*_2_^*b*^	28.00	−	−	−	−	−	−	31.01	14.638	10.8	0.51	0.905	43.25
M14	*κ*_1_	2.00	2.01	0.115	0.6	0.06	0.946	0.44	2.00	0.049	0.0	0.02	0.952	0.20
	*α*_0_	3.50	3.50	0.061	0.1	0.02	0.936	0.24	3.50	0.027	0.0	0.01	0.949	0.11
	*α*_1_	4.50	4.54	0.232	0.9	0.05	0.949	0.90	4.50	0.100	0.0	0.02	0.955	0.40
	*κ*_2_	2.50	2.55	0.391	2.1	0.15	0.951	1.47	2.52	0.164	0.8	0.07	0.952	0.64
	*θ*_2_	10.00	10.07	0.952	0.7	0.09	0.936	3.46	10.00	0.372	0.0	0.04	0.955	1.46
M15	*κ*_1_	1.50	1.50	0.080	0.1	0.05	0.948	0.30	1.50	0.035	0.0	0.02	0.953	0.14
	*θ*_1_	25.00	25.05	1.011	0.2	0.04	0.951	3.97	25.02	0.448	0.1	0.02	0.952	1.77
	*κ*_2_	1.50	1.58	0.489	5.1	0.32	0.958	1.79	1.52	0.196	1.3	0.13	0.962	0.75
	*β*_0_	3.00	3.10	0.536	3.4	0.18	0.920	1.96	3.01	0.197	0.4	0.07	0.947	0.77
	*β*_1_	1.50	1.56	0.307	4.3	0.20	0.966	1.07	1.51	0.117	0.7	0.08	0.941	0.44

PAR, parameter; MCM, Monte Carlo means; RMSE, root mean squared error; RB, relative bias % ; CV, coefficient of variation; CR, coverage rate of a Wald-based 95% confidence interval; AW, average confidence interval width.

Note: *λ*_1_ and *λ*_2_ represent the exponential rate parameters of the first and second transition times, respectively; *α*_0_ and *α*_1_ represent the regression intercept and regression coefficient of the covariate *w*∼*N*(0,1) for the first transition time; *β*_0_ and *β*_1_ represent the regression intercept and regression coefficient of the covariate *w*∼*N*(0,1) for the second transition time; *κ*_1_ and *κ*_2_ represent the Weibull shape parameters of the first and second transition times, respectively; *θ*_1_ and *θ*_2_ represent the Weibull scale parameters of the first and second transition times, respectively.

^a^The negative signs correspond to underestimation (overestimation) for positive (negative) true values while the positive signs correspond to overestimation (underestimation) for positive (negative) true values. The 0.0 values are due to approximation.

^b^The estimates were extremely large due to the small sample size and the small proportion of CRCs

Supplementary Figure S1 in the Additional file 2 shows the comparison between the true survival probability curve to those fitted using our method and the two-phase semi-Markov model by Titman and Sharples [25], averaged over 500 successful simulation runs. The two-phase semi-Markov model fits poorly for Y in both scenarios and worst in Scenario I which is a more realistic setting. Furthermore, the two-phase semi-Markov model achieved 1.3% and 4.6% successful simulation runs per 1000 replicated runs for Scenarios I and II, respectively. Our method achieved 100% successful simulation runs in both scenarios (Table 1).

### Application to the Norwegian adenoma cohort

**Fig. 4:**
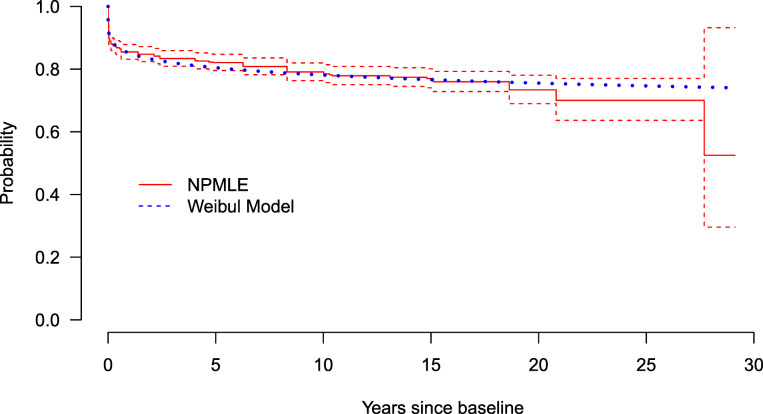
Comparison between survival curves from NPMLE estimate and Weibull model for the first transition time to AA

**Fig. 5:**
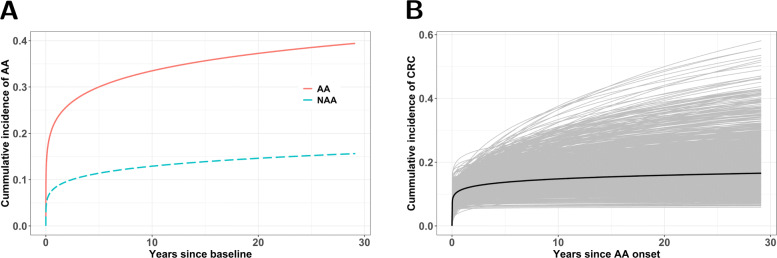
Estimated cumulative incidence curves. (**A**) Cumulative distribution function (CDF) for patients treated with AA (red solid line) and NAA (blue dashed lines) since baseline. (**B**) CDF of CRC (black solid line) since AA onset, with 1000 bootstrapped CDF curves (grey lines)

We illustrate the proposed method by fitting all 16 proposed model specifications to the Norwegian adenoma cohort described above. Table 5 describes the characteristics of the individuals in the subcohort included in the analysis during a median follow-up of 11.3 years (interquartile range 3.1;15.3 years). Parameter estimates for each of the proposed model specifications were obtained by maximizing the joint likelihood function. We performed backward stepwise regression to select variables with 5% level of significance. The resulting ML estimates, *P* values, 95*%* CIs, Akaike information criterion (AIC) values, and Bayesian information criterion (BIC) values are reported in Supplementary Table S2 in the Additional file 2. Model M14, hereafter referred to as final model, was selected as the best model based on the lowest AIC (1601.79) and BIC (1625.68) values (Table 6). To assess the goodness-of-fit of the assumed Weibull distribution for the first transition time *X* in the final model, an informal test was carried out by comparing the survival curves from the model-based estimates to the non-parametric ML estimates (NPMLEs) for interval-censored data on individuals who were observed to have developed AA (Fig. 4). The Weibull model appears to fit the data well since the curves are very close to each other. Table 6 shows that individuals who were treated for AA, have about three times the risk of having a recurrence when compared to individuals treated for NAA (hazard ratio: exp(*α*_1_)=2.95,95*%* CI: 2.18;3.98). Figure 5 depicts the cumulative incidence of AA since baseline and cumulative incidence of CRC since AA onset. Within 5 and 15 years, about 11.4*%* (95*%* CI: 8.8*%*;13.6*%*) and 13.9*%* (95*%* CI: 10.6*%*;16.6*%*), respectively, of the individuals treated for NAA at baseline will develop AA (Fig. 5a). Also, for individuals treated for AA at baseline, about 30.0*%* (95*%* CI: 25.6*%*;34.6*%*) and 35.7*%* (95*%* CI: 31.0*%*;40.6*%*) of the individuals will develop recurrent AA within 5 and 15 years, respectively (Fig. 5a). The estimates of the log shape parameter *κ*_2_ and the log scale parameter *θ*_2_ for *Y* are given in Table 6. This translates into an estimate of the shape parameter *κ*_2_ as 0.116 (95*%* CI: 0.020;0.689), indicating a decreasing hazard of CRC since onset of AA. Since we cannot observe *Y* directly, the appropriateness of the assumed Weibull distribution for *Y* can be examined, as suggested by Hudgens et al. [51], by testing *H*_0_:*κ*_2_=1 vs. *H*_*A*_:*κ*_2_≠1 using the estimates for the shape parameter *κ*_2_ above. We can see that the shape parameter *κ*_2_ is statistically different from one at 5*%* level of significance. The bootstrapped curves in Fig. 5b show there was considerable uncertainty about the cumulative incidence of CRC from AA; with the uncertainty increasing with time. This was largely due to small sample size and low proportion of CRCs in the data. Within 5 and 15 years, about 13.8*%* (95*%* CI: 7.8*%*;23.8*%*) and 15.4*%* (95*%* CI: 8.2*%*;34.0*%*) of the individuals will develop CRC, respectively. The mean time among those who have had the transition to CRC since AA onset within 50 years was estimated to be 4.80 years (95% CI: 0; 7.61) using a right-truncated Weibull distribution [52].

**Table 5:** Patient characteristics of the Norwegian adenoma cohort used for analysis

	Finding at follow-up			
Characteristics	AF (***n=688***)	AA (***n=170***)	CRC (***n=20***)	Total (***n=878***)
*%* of total	78.4	19.4	2.2	100
Age, yr, mean (SD)	65.0 (11.5)	64.6 (10.2)	70.0 (10.1)	65.0 (11.2)
Sex (*%*)				
Male	342 (49.7)	79 (46.5)	11 (55.0)	432 (49.2)
Female	346 (50.3)	91 (53.5)	9 (45.0)	446 (50.8)
AT (*%*)				
AA	271 (39.4)	113 (66.5)	14 (70)	398 (54.7)
NAA	417 (60.6)	57 (33.5)	6 (30.0)	480 (45.3)
FH (*%*)				
Yes	84 (12.2)	27 (15.9)	1 (5.0)	112 (12.8)
No	604 (87.8)	143 (84.1)	19 (95.0)	766 (87.2)

AF, adenoma-free; AT, adenoma-type; NAA, non-advanced adenoma; AA, advanced adenoma; CRC, colorectal cancer; FH, Family history (First degree relatives with CRC).

**Table 6:** Result of the final model of the Norwegian adenoma cohort

Transition	Distribution	Parameter	Estimate	***P*** value	***95%*** CI
First (*X*)	Weibull	shape, log(*κ*_1_)	− 1.646	< 0.001	(−1.849;−1.442)
		Intercept, *α*_0_	12.561	< 0.001	(10.117;15.004)
		AT: AA, *α*_1_	1.081	< 0.001	(0.780;1.382)
Second (*Y*)	Weibull	shape, log(*κ*_2_)	− 2.153	0.02	(−3.935;−0.372)
		scale, log(*θ*_2_)	18.087	0.29	(−15.382;51.555)

AT, adenoma-type; AA, advanced adenoma

## Discussion

In this paper, we proposed a modeling framework to jointly estimate both the transition time *X* from AF to AA and the transition time *Y* from AA to CRC based on CRC surveillance data using a progressive three-state disease model. The reliability of the method was shown by simulation studies and was illustrated using a Norwegian adenoma cohort. Our simulation results show that the estimates of the parameters of the time distributions are consistent and the 95*%* confidence intervals have good coverage.

### Modeling framework

Our proposed method distinguishes itself from other methods used for estimating the time distributions in a progressive three-state disease model in that our method provides a likelihood function that in principle, accommodates any probability distribution without covariates (see **R** implementation code in the Additional file 1), is based on surveillance data that are interval-censored for both transition times, allows the inclusion of covariates at both times, and models a disease process where an individual is censored once the second health state is observed to occur. A comparison between our method and the two-phase semi-Markov model by Titman and Sharples [25] via simulation showed that our method is more accurate and stable when handling data arising from disease processes where state 2 to 3 is never observed; contrary to the observation process in the model by Titman and Sharples [25] where transition to state 3 is exactly known. These results demonstrate the importance of using our tailored method for modeling surveillance data.

We focused on PH models with the exponential and Weibull distributions as an illustration, but also the Gompertz distribution can be used and was implemented in the **R** code in the Additional file 1. Inclusion of covariates on both *X* and *Y* allows capturing the dependency between both transition times and examining possible population heterogeneity.

Our proposed method is based on the maximum likelihood framework, which can encounter problems during estimation. First, numerical issues can be encountered when performing the optimization or numerical integration of the probability expressions in the likelihood function. A few of the issues include divergence of the integrand and the so-called *false convergence*, a situation where the optimization algorithm reports a solution (i.e., convergence) but the Hessian matrix which is needed for computing the SE of the model parameters fails to be positive definite. These issues could be solved by adjusting some of the default settings of the integrate() and optim() functions. Second, the stability of the optimization algorithm is fairly dependent on the choice of the starting values and the nature of the likelihood surface (i.e., unimodal, flat, or multimodal), particularly for more complex models. Hence, it is recommended to run complex models using different starting values to ensure that the optimization algorithm successfully converged to global optima (i.e., true ML estimates) instead of local optima. Similar observations have been made in the literature [23, 25, 53].

### Application to CRC surveillance

Yen et al. [30] noted that quantification of heterogeneity by identifying risk groups or factors associated with rapid progression to AA or to CRC since the onset of AA is an important step in determining the potential value of personalizing surveillance intervals. Analysis of the Norwegian adenoma cohort showed that individuals who were treated for AA at baseline have about three times higher risk of developing an AA when compared to individuals who were treated for NAA instead. This is expected. Similar findings have been reported before [54, 55]. For example, our finding is in agreement with Laiyemo et al. [55], who reported a relative risk of AA recurrence in individuals with high- versus low-risk adenomas at baseline of 1.68 (95% CI: 1.19; 2.38). These findings are the reason for current more intensive surveillance recommendations after AA removal compared to NAA removal [6, 54]. The difference in risk to progress to AA between individuals in whom an AA was removed versus those with NAA removed could also possibly hold for the transition from AA to CRC. However, this was not estimated in our final model. The lack of significance of the adenoma-type variable in the second transition time may have been the result of the small sample size, particularly the small number of CRCs, in the current study. A large amount of data is needed to substantiate our hypothesis, especially with respect to CRC cases.

For the transition from AA to CRC, we found that around 15% of individuals will develop CRC within 15 years after AA onset. Note that about 10% of these individuals developed a CRC at the same time or even earlier than the average time it takes to develop an AA from baseline (Fig. 5b). This suggests that some of the AA cases are rapidly progressing and there is likely substantial heterogeneity in duration between individuals. However, there is considerable degree of uncertainty in the CRC cumulative incidence. This is in part because of the relatively small sample size and in particular the low proportion of CRC cases. Another reason, as shown in the simulation studies, is the inherent uncertainty that is always associated with estimating Y. Estimates of the cumulative risk or average time to CRC since adenoma onset have previously been published [14, 16, 30, 56]. For instance, Brenner et al. [16] studied the age and sex-specific risk of CRC from AA onset using data from a nationwide registry of screening colonoscopies in Germany. At age 55 years, the 10-year cumulative risk for both sexes was estimated to be around 25%. Cafferty et al. [57] and Yen et al. [30] showed that within 20 years, 57% and 40% of adenomas (of any type), respectively, will progress to CRC. We found the hazard from AA to CRC to be decreasing with time since onset of AA, again suggesting heterogeneity in risk, with fast and slow transforming lesions. There is some evidence for such differences in malignant potential among AAs, based on molecular characterization of adenomas [58]. Surveillance might not be warranted for those individuals with indolent or slow transforming lesions as this would lead to overdiagnosis, but there is currently no solid means to identify these individuals. This time distribution from AA to CRC is a key parameter in explaining differences in the outcomes of microsimulation models used for the optimization of CRC prevention [13, 59]. Our method allows for improving these models by the inclusion of flexible statistical time distributions rather than using expert assumptions or model calibration. Surveillance intervals are currently recommended based on the outcome of examination findings and the risk status of an individual [6, 54]. For instance, the general consensus is that a 10-year interval for colonoscopy should be recommended for average-risk individuals [6, 54]. Such time interval has not been determined in a systematic way. We suggest that recommendations for screening and surveillance intervals should be based on the rate of transitioning of the disease, as also stated by Frame and Frame [60]. Our modelling framework provides such estimates. Our projections of cumulative incidence of CRC allows one to predict the number of CRC cases that would have developed if there would not have been any surveillance and newly developing AA would not be detected and removed. We estimated that about 14% of AA cases will develop to CRC within 5 years and that only an additional 1% will develop to CRC in the subsequent 10 years. This suggests that 10 years follow-up surveillance after the initial 5 years may not necessarily yield any added benefit, and only short-term surveillance is required. We also estimated the mean time among those who have made the transition to CRC since AA onset within 50 years to be 4.80 years (95% CI: 0; 7.61). Thus, indicating the need for short-term surveillance due to the short amount of time it may take to progress to CRC from AA.

### Future directions

We assumed that every individual having an AA has the potential to develop CRC. As noted by Van Ballegooijen et al. [13] and Lew at al. [61], vast majority of individuals with AA will not develop a CRC in their lifetime and diagnosis and treatment of these AA could be seen as overdiagnosis. To account for the possibility of a difference in risk, *Y* could be modelled as a mixture distribution accounting for individuals with progressive versus indolent lesions [19, 62] or by explicitly modelling the dependence between *X* and *Y* since fast progression from AF to AA maybe followed by fast progression from AA to CRC as noted similarly in the cervical cancer model proposed by Vink et al. [19]. Furthermore, we assumed that at baseline, after polypectomy, all individuals are adenoma free. This assumption could be relaxed to allow for the possibility that a small proportion of non-advanced or advanced adenomas, or even CRC, are missed at colonoscopy.

## Conclusion

Reliable estimation of the time distribution between precancer and cancer is important to allow prediction of long-term outcomes of screening and surveillance programs and to allow optimization of such programs. We have provided a statistical method for estimating the not directly-observable time from AA to CRC in a progressive three-state disease model. Our proposed method is not limited to estimating time distributions in the CRC screening and surveillance setting, but can be applied to any disease process where individuals are censored once they are observed to be in a pre-final state and are treated in that disease state, such that the progress from a pre-final to final state cannot be observed.

## Supplementary Information

**Additional file 1** R implementation codes.

**Additional file 2** Supplementary Material.

## Data Availability

De-identified data that support the finding of this study may be available from Henriette C. Jodal (h.c.jodal@medisin.uio.no), however restrictions apply to the availability of these data, and permission of sharing may be subject to approval by the Regional Research Ethics Committee of South-Eastern Norway.

## Abbreviations

CRC: Colorectal Cancer
AA: Advanced Adenoma
AF: Adenoma-Free
HPV: Human Papillomavirus
PH: Proportional Hazards
PDFs: Probability Density Functions
CDFs: Cumulative Distribution Functions
ML: Maximum Likelihood
MC: Monte Carlo
RMSE: Root Mean Squared Error
RB: Relative Bias
CV: Coverage Variability
SE: Standard Error
CR: Coverage Rate
CI: Confidence Interval
AW: Average Width
AT: Adenoma-Type
NPMLEs: Non-Parameteric Maximum Likelihood Estimates
AIC: Akaike Information Criterion
BIC: Bayesian Information Criterion
