# Supplementary material for: A progressive three-state model to estimate time to cancer: a likelihood-based approach
Source: BMC Med Res Methodol. 2022 Jun 27;22:179. doi: 10.1186/s12874-022-01645-2 (PMC9235269; doi:10.1186/s12874-022-01645-2)
Supplement: Supplementary file 1 — Additional file 1 R implementation codes. [file 12874_2022_1645_MOESM1_ESM.pdf]

Additional file 1: R implementation codes

This file contains the R codes used for simulation in the main paper. The following model specifications with their respective parameters in parentheses are implemented to show the main focus of the paper (i.e., parametric proportional hazards models):

1. **Exp**onential distribution without covariate for both X (`X.rate`) and Y (`Y.rate`).
2. **Exp**onential distribution with covariate for both X (`alpha0, alpha1`) and Y (`beta0, beta1`).
3. **Weibull** distribution without covariate for both X (`X.scale, X.shape`) and Y (`Y.scale, Y.shape`).
4. **Weibull** distribution with covariate for both X (`X.shape, alpha0, alpha1`) and Y (`Y.shape, beta0, beta1`).
5. **Gompertz** distribution without covariate for both X (`X.scale, X.shape`) and Y (`Y.scale, Y.shape`).
6. **Gompertz** distribution with covariate for both X (`X.shape, alpha0, alpha1`) and Y (`Y.shape, beta0, beta1`).

To demonstrate that our method accommodates other survival probability distributions without covariate, the following model specifications with their respective parameters in parentheses are implemented as well:

7. **Loglogisti**c distribution without covariate for both X (`X.scale, X.shape`) and Y (`Y.scale, Y.shape`).
8. **Lognormal** distribution without covariate for both X (`X.meanlog, X.sdlog`) and Y (`Y.meanlog, Y.sdlog`).
9. **Gamma** distribution without covariate for both X (`X.scale, X.shape`) and Y (`Y.scale, Y.shape`).

While loglogistic, lognormal, and Gamma probability distributions are used in accelerated failure time models as well, i.e., when examining covariate effects, the implementation of these 3 distributions with covariates is not the focus of the paper as mentioned in the *Discussion* section of the main manuscript. Detailed description of each of the above 6 survival probability distributions is provided in the book by Collett [1].

The simulation codes contain the following 7 **R** files which needs to be **copied and save** in the same folder of the user's computer:

1. `simDatax.R`: This file contains the `dat()` function and it is used to simulate right-,left-, and interval-censored data.

2. `probExpressions.R`: This file contains the probability expressions for all the model specifications mentioned above.

3. `likelihood.R`: This file contains the log-likelihood functions (L1 to L9) for the 9 models specifications above.

4. `maxmfun.R`: This file contains the `maxmfun2()` function that performs the maximization process using `optim()` function in **R** with the maximum likelihood (ML) estimates and the corresponding standard error as outputs.

5. `fxn.R`: This file contains the `cal()` function that computes the performance measures defined in the "Simulation Studies" section of the main paper and the percentage of successful simulation runs.

6. `simDemo1.R`: This is the **main** file which contains all the above **R** files and is used for demonstrating the maximization process for a single run.

7. `simDemo2.R`: This file is for demonstrating the Monte Carlo simulation with the performance measures as output. The number of simulation used in the main paper is $Nsim = 1000$. Increasing the number of simulation, increases the computational time.

Demonstration Example:

A Weibull model without covariate for both X (`X.scale = 15.0 , X.shape = 1.5`) and Y (`Y.scale = 5.0, Y.shape = 0.8`) is included as an example in both the `simDemo1.R` and `simDemo2.R` files.
To run this example model for a single simulation, please run the `simDemo1.R` code and to perform a Monte Carlo simulation for 1000 iterations, please run the `simDemo2.R` code.

1 simData.R

```
library(eha)

dat = function(n,                # Number of individuals; the larger the sample
    size, the more consistent the estimates
X.dist  = NULL,   # Distribution of first transition X; options are Exp, Weibull ,
    Gompertz, Loglogistic, Lognormal & Gamma
Y.dist  = NULL,   # Distribution of second transition Y; options are Exp, Weibull ,
    Gompertz, Loglogistic, Lognormal & Gamma
alpha0  = NULL,   # Regression intercept for first transition X
alpha1  = NULL,   # Regression coefficient of covariate W for first transition X
beta0   = NULL,   # Regression intercept for second transition Y
beta1   = NULL,   # Regression coefficient of covariate W for second transition Y
X.rate  = NULL,   # Exponential rate parameter for first transition X
Y.rate  = NULL,   # Exponential rate parameter for second transition Y
X.shape = NULL,   # Weibull, Gompertz,  Loglogistic & Gamma shape parameter for
    first transition X
Y.shape = NULL,   # Weibull, Gompertz,  Loglogistic & Gamma shape parameter  for
    second transition Y
X.scale = NULL,   # Weibull, Gompertz,  Loglogistic & Gamma scale parameter for
    first transition X
Y.scale = NULL,   # Weibull, Gompertz,  Loglogistic & Gamma scale parameter for
    second transition Y
X.meanlog   =    NULL,  # Lognormal mean parameter for first transition X
X.sdlog     =    NULL,  # Lognormal standard deviation parameter for first
    transition X
Y.meanlog   =    NULL,  # Lognormal mean parameter for second transition Y
Y.sdlog     =    NULL,  # Lognormal standard deviation parameter for second
    transition Y
p.X,              # covariate for first transition X? Two options: 1 = yes & 0 = No
p.Y              # covariate for second transition Y? Two options: 1 = yes & 0 = No
){


  W = rnorm(n)          # standard normal distributed covariate

  if( X.dist == 'Exp' &  Y.dist == 'Exp'){
    # Exponential and Exponential model without covariate
    if(p.X ==0&p.Y ==0){
      X.rate = X.rate
      Y.rate = Y.rate
      # Exponential + covariate and Exponential + covariate model
    } else{
      X.rate   =  exp(alpha0 + W* alpha1 )
      Y.rate   =  exp(beta0+ W * beta1)
    }

    X          =  rexp(n,rate = X.rate)                    # time from AF to AA

    Y          =  rexp(n,rate = Y.rate)                    # time from AA to CRC

  }else if( X.dist == 'Weibull' &  Y.dist == 'Weibull'){
    # Weibull and Weibull model without covariate
    if(p.X ==0&p.Y ==0){
      X.shape   =   X.shape
```

```
    Y.shape    =   Y.shape
    X.scale    =   X.scale
    Y.scale    =   Y.scale
    # Weibull + covariate  and Weibull + covariate  model
  } else{
    X.shape    =   X.shape
    Y.shape    =   Y.shape
    X.scale    =   exp(alpha0 - (W* alpha1 )/X.shape )
    Y.scale    =   exp(beta0 -  (W * beta1)/Y.shape)
  }

  X            =  rweibull(n, shape = X.shape, scale = X.scale)  # time from AF to
  AA

  Y            =  rweibull(n, shape = Y.shape, scale = Y.scale)  # time from AA to
  CRC
}

else if( X.dist == 'Gompertz' &  Y.dist == 'Gompertz'){
  # Gompertz and Gompertz model without covariate
  if(p.X ==0&p.Y ==0){
    X.shape    =   X.shape
    Y.shape    =   Y.shape
    X.scale    =   X.scale
    Y.scale    =   Y.scale
    # Gompertz + covariate and Gompertz + covariate model
  } else{
    X.shape   =   X.shape
    Y.shape   =   Y.shape
    X.scale   =   exp(alpha0 + W* alpha1)
    Y.scale   =   exp(beta0 +  W * beta1)
  }

  X           =  eha::rgompertz(n, shape = X.shape, scale  = X.scale)  # time from
  AF to AA

  Y           =  eha::rgompertz(n, shape = Y.shape, scale = Y.scale)   # time from
  AA to CRC
}
else if( X.dist == 'Loglogistic' &  Y.dist == 'Loglogistic'){
  # Loglogistic and Loglogistic model without covariate
  if(p.X ==0&p.Y ==0){
    X.shape    =   X.shape
    Y.shape    =   Y.shape
    X.scale    =   X.scale
    Y.scale    =   Y.scale

  } else{
    stop('Define p.X==0 and p.Y==0 for Loglogistic instead')
  }

  X           =  eha::rllogis(n, shape = X.shape, scale  = X.scale)  # time from
 AF to AA

  Y           =  eha::rllogis(n, shape = Y.shape, scale = Y.scale)   # time from
 AA to CRC
}
```

```
else if( X.dist == 'Lognormal' &  Y.dist == 'Lognormal'){
  #Lognormal and Lognormal model without covariate
  if(p.X ==0&p.Y ==0){
    X.meanlog   =    X.meanlog
    X.sdlog     =    X.sdlog
    Y.meanlog   =    Y.meanlog
    Y.sdlog     =    Y.sdlog

  } else{
    stop('Define p.X==0 and p.Y==0 for Lognormal instead')
  }

  X             =    rlnorm(n=n,meanlog =X.meanlog,sdlog = X.sdlog )  # time from
   AF to AA

  Y             =    rlnorm(n=n,meanlog =Y.meanlog,sdlog = Y.sdlog )   # time
 from AA to CRC
}
else if( X.dist == 'Gamma' &  Y.dist == 'Gamma'){
  # Gamma and Gamma model without covariate
  if(p.X ==0&p.Y ==0){
    X.shape   =   X.shape
    Y.shape   =   Y.shape
    X.scale   =   X.scale
    Y.scale   =   Y.scale

  } else{
    stop('Define p.X==0 and p.Y==0 for Gamma instead')
  }

  X          =  rgamma(n, shape = X.shape, scale  = X.scale)  # time from AF to
 AA

  Y          =  rgamma(n, shape = Y.shape, scale = Y.scale)   # time from AA to
 CRC
}
else{
  stop('Check your model specifications')
}


# create an empty matrix to store the visits when an individual is observed to be
   in AF, AA or CRC states
r    =    matrix(NA,nr=n,nc=4,byrow = FALSE)


Z    =    X  +  Y   # total time from AF to CRC

XZ   =    cbind(X,Z)  # time from HE to AA  &  time from HE to CRC

# follow-up visit times for all patients ( max of 4 visits)
I    =    as.data.frame(t(sapply(1:n,function(b) sort(sample(1:20,4,replace =
 FALSE)) )))

#  Compare time to AA with follow-up visit times
for(i in 1:nrow(r)){
  if(XZ[i,1]<=I[i,1] & XZ[i,2]>I[i,1]){
```

```
    r[i,1] <- 1
  }else if(XZ[i,1]<=I[i,2] & XZ[i,2]>I[i,2]){
    r[i,2] <- 1
  }else if(XZ[i,1]<=I[i,3] & XZ[i,2]>I[i,3]){
    r[i,3] <- 1
  }else if(XZ[i,1]<=I[i,4] & XZ[i,2]>I[i,4]){
    r[i,4] <- 1
  }else{
    r[i,] <- 0
  }
}

#  Compare time to CRC with follow-up visit times
for(i in 1:nrow(r)){
  if(XZ[i,1]<I[i,1] & XZ[i,2]<=I[i,1] & !any(r[i,]==1)   ){
    r[i,1] <- 2
  }else if(XZ[i,1]<I[i,2] & XZ[i,2]<=I[i,2] & !any(r[i,]==1)){
    r[i,2] <- 2
  }else if(XZ[i,1]<I[i,3] & XZ[i,2]<=I[i,3] & !any(r[i,]==1)){
    r[i,3] <- 2
  }else if(XZ[i,1]<I[i,4] & XZ[i,2]<=I[i,4] & !any(r[i,]==1)){
    r[i,4] <- 2
  }
}


# matrix r contains health states of individuals (0= AF, 1= AA, 2 = CRC)
r =  as.data.frame(r)


# Obtain  left-, right-, and interval censored data  for individuals

r.af = as.numeric(row.names( subset(r,rowSums(r)==0)))

C.af  = t(sapply(r.af, function(g) cbind(g,4,0) ) )
C.aa =  cbind(which(r==1,arr.ind = T), 1)
C.crc = cbind(which(r==2,arr.ind = T),2)

H = rbind(C.af,C.aa,C.crc)

D = matrix(nr= nrow(H),nc=3)

for(h in seq_len(nrow(H))){

  dd = H[h,]
  if(dd[2]==1) D[h,] = c(0, I[dd[1], dd[2]], dd[3]) else D[h,] = c( I[dd[1], dd
 [2]-1] , I[dd[1], dd[2]], dd[3])


}

D = as.data.frame(D)

# a and b are left and right points of the interval-censored data, respectively

# event means health state (0= AF, 1 = AA, 2= CRC)
names(D) = c('a','b','event')
```

```
  W = W[H[,1]]   # extract covariates

  if(p.X ==0& p.Y==0) data.X =  D  else data.X =  cbind(D,W)

  return(  data.X )

}
```

2 probExpressions.R

```
library(eha)


# Analytic probability expressions for an exponential model for both X and Y. See
   Appendix C.1

p.aa = function(theta, lambda, a, b){
  return(ifelse(theta==lambda, log(theta)+log(b-a)-theta*b, log( (theta/(theta-
  lambda))*(exp(-theta*a-lambda*(b-a)) - exp(-b*theta)) ) ))
}
p.aa=Vectorize(p.aa)
p.crc = function(theta, lambda, a, b){
  return(ifelse( theta==lambda,-theta*b+ log( exp(-theta* (a-b)) - theta*(b-a)-1
  ),-theta*a + log( abs(theta/(lambda-theta) * exp(-lambda*(b-a)) - lambda/(lambda
  -theta) * exp(-theta*(b-a)) +1)) ))
}
p.crc=Vectorize(p.crc)
p.af = function(theta, b){
  -theta*b
}
p.af=Vectorize(p.af)



# Probability expressions for a Weibull model for both X and Y using formula in
  Appendix A
int1 = function(x,b,w.sc1,w.sh1,w.sc2,w.sh2){dweibull(x,shape = w.sh1,scale = w.
  sc1)*pweibull(b-x,shape = w.sh2,scale = w.sc2,lower.tail = FALSE)}
paa = function(l,u,w.sc1,w.sh1,w.sc2,w.sh2){ abs(integrate(int1,lower=l,upper=u,b
  =u,w.sh1=w.sh1,w.sc1= w.sc1,
   w.sh2=w.sh2,w.sc2= w.sc2,stop.on.error = FALSE)$value)}
paa=Vectorize(paa)

int2 = function(x,b,w.sc1,w.sh1,w.sc2,w.sh2){dweibull(x,shape = w.sh1,scale = w.
  sc1)*pweibull(b-x,shape = w.sh2,scale = w.sc2)}
pcc = function(l,u,w.sc1,w.sh1,w.sc2,w.sh2){ abs(integrate(int2,lower=l,upper=u,b
  =u,w.sh1=w.sh1,w.sc1= w.sc1,
   w.sh2=w.sh2,w.sc2= w.sc2,stop.on.error = FALSE)$value)}
pcc  =   Vectorize(pcc)

paff =  function(w.sc1,w.sh1, u){
  pweibull(u,shape = w.sh1,scale = w.sc1,lower.tail = FALSE,log.p = TRUE)
}
```

```
paff=Vectorize(paff)



# Probability expressions for a Gompertz model for both X and Y using formula in
  Appendix A
int3 = function(x,b,g.sc1,g.sh1,g.sc2,g.sh2){eha::dgompertz(x,shape = g.sh1,scale
  = g.sc1)*eha::pgompertz(b-x,shape = g.sh2,scale = g.sc2,lower.tail = FALSE)}
pa = function(l,u,g.sc1,g.sh1,g.sc2,g.sh2){ abs(integrate(int3,lower=l,upper=u,b=
 u,g.sh1=g.sh1,g.sc1= g.sc1,
  g.sh2=g.sh2,g.sc2= g.sc2,stop.on.error = FALSE)$value)}
pa=Vectorize(pa)

int4 =  function(x,b,g.sc1,g.sh1,g.sc2,g.sh2){eha::dgompertz(x,shape = g.sh1,
 scale = g.sc1)*eha::pgompertz(b-x,shape = g.sh2,scale = g.sc2)}
pc =  function(l,u,g.sc1,g.sh1,g.sc2,g.sh2){ abs(integrate(int4,lower=l,upper=u,b
 =u,g.sh1=g.sh1,g.sc1= g.sc1,
  g.sh2=g.sh2,g.sc2= g.sc2,stop.on.error = FALSE)$value)}
pc=Vectorize(pc)

paf =  function(g.sc1,g.sh1, u){
  eha::pgompertz(u,shape = g.sh1,scale = g.sc1,lower.tail = FALSE,log.p = TRUE)

}
paf= Vectorize(paf)


# Probability expressions for a loglogistic model for both X and Y using formula
 in  Appendix A
int5 = function(x,b,ll.sc1,ll.sh1,ll.sc2,ll.sh2){eha::dllogis(x,shape = ll.sh1,
 scale = ll.sc1)*eha::pllogis(b-x,shape = ll.sh2,scale = ll.sc2,lower.tail =
 FALSE)}
pAa = function(l,u,ll.sc1,ll.sh1,ll.sc2,ll.sh2){ abs(integrate(int5,lower=l,upper
 =u,b=u,ll.sh1=ll.sh1,ll.sc1= ll.sc1,
  ll.sh2=ll.sh2,ll.sc2= ll.sc2,stop.on.error = FALSE)$value)}
pAa=Vectorize(pAa)

int6 =  function(x,b,ll.sc1,ll.sh1,ll.sc2,ll.sh2){eha::dllogis(x,shape = ll.sh1,
 scale = ll.sc1)*eha::pllogis(b-x,shape = ll.sh2,scale = ll.sc2)}
pcRc = function(l,u,ll.sc1,ll.sh1,ll.sc2,ll.sh2){ abs(integrate(int6,lower=l,
 upper=u,b=u,ll.sh1=ll.sh1,ll.sc1= ll.sc1,
  ll.sh2=ll.sh2,ll.sc2= ll.sc2,stop.on.error = FALSE)$value)}
pcRc=Vectorize(pcRc)

paF =  function(ll.sc1,ll.sh1, u){
  eha::pllogis(u,shape = ll.sh1,scale = ll.sc1,lower.tail = FALSE,log.p = TRUE)

}
paF = Vectorize(paF)


# Probability expressions for a lognormal model for both X and Y using formula in
   Appendix A

int7 =  function(x,b,sdlog1,meanlog1,sdlog2,meanlog2){dlnorm(x,meanlog = meanlog1
 ,sdlog = sdlog1)*plnorm(b-x,meanlog = meanlog2,sdlog = sdlog2,lower.tail = FALSE
```

```
  )}
p.Aa =  function(l,u,sdlog1,meanlog1,sdlog2,meanlog2){ abs(integrate(int7,lower=l
 ,upper=u,b=u,meanlog1=meanlog1,sdlog1= sdlog1,
  meanlog2=meanlog2,sdlog2= sdlog2,stop.on.error = FALSE)$value)}
p.Aa  = Vectorize(p.Aa )

int8 = function(x,b,sdlog1,meanlog1,sdlog2,meanlog2){dlnorm(x,meanlog = meanlog1,
 sdlog = sdlog1)*plnorm(b-x,meanlog = meanlog2,sdlog = sdlog2)}
p.cRc  =  function(l,u,sdlog1,meanlog1,sdlog2,meanlog2){ abs(integrate(int8,lower
 =l,upper=u,b=u,meanlog1=meanlog1,sdlog1= sdlog1,
  meanlog2=meanlog2,sdlog2= sdlog2,stop.on.error = FALSE)$value)}
p.cRc = Vectorize(p.cRc)

p.aF  =  function(sdlog1,meanlog1, u){
  plnorm(u,meanlog = meanlog1,sdlog = sdlog1,lower.tail = FALSE,log.p = TRUE)

}
p.aF  = Vectorize(p.aF )


# Probability expressions for a Gamma model for both X and Y using formula in
 Appendix A


int9 =  function(x,b,ga.sc1,ga.sh1,ga.sc2,ga.sh2){dgamma(x,shape = ga.sh1,scale =
  ga.sc1)*pgamma(b-x,shape = ga.sh2,scale = ga.sc2,lower.tail = FALSE)}
p.a =  function(l,u,ga.sc1,ga.sh1,ga.sc2,ga.sh2){ abs(integrate(int9,lower=l,
 upper=u,b=u,ga.sh1=ga.sh1,ga.sc1= ga.sc1,
  ga.sh2=ga.sh2,ga.sc2= ga.sc2,stop.on.error = FALSE)$value)}
p.a=Vectorize(p.a)

int10 = function(x,b,ga.sc1,ga.sh1,ga.sc2,ga.sh2){dgamma(x,shape = ga.sh1,scale =
  ga.sc1)*pgamma(b-x,shape = ga.sh2,scale = ga.sc2)}
p.c =  function(l,u,ga.sc1,ga.sh1,ga.sc2,ga.sh2){ abs(integrate(int10,lower=l,
 upper=u,b=u,ga.sh1=ga.sh1,ga.sc1= ga.sc1,
  ga.sh2=ga.sh2,ga.sc2= ga.sc2,stop.on.error = FALSE)$value)}
p.c=Vectorize(p.c)

p.f  =  function(ga.sc1,ga.sh1, u){
  pgamma(u,shape = ga.sh1,scale = ga.sc1,lower.tail = FALSE,log.p = TRUE)

}
p.f = Vectorize(p.f)
```

3 likelihood.R

```
library(tidyverse)
# The following defines the log-likelihood function for the following model
   specifications:

# Exponential and Exponential
L1 = function(pars, data=data.X) {
  data.af   =   subset(data,event==0,select=c(a,b))              # AF  individuals
```

```
  data.aa    =   subset(data,event==1,select=c(a,b))              # AA individuals
  data.crc   =   subset(data,event==2,select=c(a,b))              # CRC individuals

  para       =   exp(pars)
  theta      =   para[1]
  lambda     =   para[2]

  lik.af     =   sum(p.af(theta=theta, b=data.af[,2]))
      # log-likelihood contribution of AF individuals
  lik.aa     =   sum(p.aa(a=data.aa[,1], b=data.aa[,2],theta=theta,lambda=lambda))
      # log-likelihood contribution of AA individuals
  lik.crc    =   sum(p.crc(a=data.crc[,1], b=data.crc[,2],theta=theta,lambda=lambda)
   ) # log-likelihood contribution of CRC individuals
  log.lik    =   c(lik.af,lik.aa,lik.crc)

  log.lik    =   sum(log.lik)
  return(-log.lik)
}


# Exponential + Covariate and Exponential + Covariate
L2 = function(para, data=data.X) {
  data.af    =  subset(data,event==0,select=c(a,b))
  data.aa    =  subset(data,event==1,select=c(a,b))
  data.crc   =  subset(data,event==2,select=c(a,b))

  X          =  select(data,c(W))                                 # Covariate
   for first transition X
  X1         =  data.matrix(cbind(1,X))
  beta.X     =  data.matrix(para[1:ncol(X1)])
  theta      =  exp(X1 %*%beta.X)

  Y          =  select(data,c(W))                                   # Covariate
   for second transition Y
  Y1         =  data.matrix(cbind(1,Y))
  beta.Y     =  data.matrix(para[(ncol(Y1)+1):(2*ncol(Y1))])
  lambda     =  exp(Y1 %*%beta.Y)

  lik.af     =  p.af(theta=theta[data$event==0,], b=data.af[,2])
  lik.aa     =  p.aa(theta=theta[data$event==1,], lambda=lambda[data$event==1,], a=
   data.aa[,1], b= data.aa[,2])
  lik.crc    =  p.crc(theta=theta[data$event==2,], lambda=lambda[data$event==2,], a=
   data.crc[,1], b=data.crc[,2])
  log.lik    =  sum(c(lik.af,lik.aa,lik.crc))
  return(-log.lik)
}

# Weibull and Weibull
L3 = function(para, data=data.X) {
  data.af    =  subset(data,event==0,select=c(a,b))
  data.aa    =  subset(data,event==1,select=c(a,b))
  data.crc   =  subset(data,event==2,select=c(a,b))

  scale.X    =  exp(para[1])                                      # Scale
   parameter for first transition X
  shape.X    =  exp(para[2])                                      # Shape
   parameter for first transition X
```

```
  scale.Y    =  exp(para[3])                                   # Scale
   parameter for second transition Y
  shape.Y    =  exp(para[4])                                   # Shape
   parameter for second transition Y
  lik.af     =  sum(paff(w.sc1=scale.X,w.sh1=shape.X, u=data.af[,2]))
  lik.aa     =  sum(log(paa( l=data.aa[,1], u=data.aa[,2],w.sc1= scale.X,w.sh1=shape
   .X,w.sc2= scale.Y,w.sh2=shape.Y)))
  lik.crc    =  sum(log(pcc( l=data.crc[,1], u=data.crc[,2],w.sc1= scale.X,w.sh1=
   shape.X,w.sc2=scale.Y,w.sh2=shape.Y)))
  log.lik    =  sum(lik.af,lik.aa,lik.crc)
  return(-log.lik)
}

# Weibull + Covariate and Weibull + Covariate
L4 = function(para, data=data.X) {

  data.af    =  subset(data,event==0,select=c(a,b))
  data.aa    =  subset(data,event==1,select=c(a,b))
  data.crc   =  subset(data,event==2,select=c(a,b))
  w.sh.X     =  as.matrix(rep(exp(para[1]),nrow(data)))            # Shape
   parameter for first transition X
  X          =  select(data,c(W))

  X1         =  data.matrix(cbind(1,-X/w.sh.X))
  beta.X     =  data.matrix(para[2:(ncol(X1)+1)])
  w.sc.X     =  exp(X1 %*%beta.X)                                   # Scale
   parameter for first transition X
  w.sh.Y     =  as.matrix(rep(exp(para[(ncol(X1)+2)]),nrow(data)))   # Shape
   parameter for second transition Y

  Y          =  select(data,c(W))


  Y1         =  data.matrix(cbind(1,-Y/w.sh.Y))
  beta.Y     =  data.matrix(para[(ncol(X1)+3):( ncol(X1)+1 + ncol(Y1)+1  ) ])
  w.sc.Y     =  exp(Y1 %*%beta.Y)                                     # Scale
   parameter for second transition Y

  lik.af     =  paff(w.sc1 = w.sc.X[data$event==0,],w.sh1=w.sh.X[data$event==0,], u=
   data.af[,2])
  lik.aa     =  log( paa(l= data.aa[,1], u= data.aa[,2],w.sc1=w.sc.X[data$event
   ==1,],w.sh1=w.sh.X[data$event==1,],
  w.sc2=w.sc.Y[data$event==1,], w.sh2=w.sh.Y[data$event==1,]))
  lik.crc    =  log( pcc(l= data.crc[,1], u= data.crc[,2],w.sc1=w.sc.X[data$event
   ==2,],w.sh1=w.sh.X[data$event==2,],
  w.sc2=w.sc.Y[data$event==2,], w.sh2=w.sh.Y[data$event==2,]))
  log.lik    =  sum(c(lik.af,lik.aa,lik.crc))
  return(-log.lik)
}



# Gompertz and Gompertz
L5 = function(para, data=data.X) {
  data.af    =  subset(data,event==0,select=c(a,b))
  data.aa    =  subset(data,event==1,select=c(a,b))
  data.crc   =  subset(data,event==2,select=c(a,b))
```

```
  scale.X    =  exp(para[1])
  shape.X    =  exp(para[2])
  scale.Y    =  exp(para[3])
  shape.Y    =  exp(para[4])

  lik.af     =  sum(paf(g.sc1=scale.X,g.sh1=shape.X, u=data.af[,2]))

  # The likelihood contribution for some AA and CRC individuals could be zero as a
   result of numeric  underflow that is
  # associated with the Gompertz distribution (due to the two exponent terms in the
    Gompertz distribution  formula for large values).
  # A quick and dirty trick is to replace such individuals with the max likelihood
   contribution value.

  ll.aa      =  pa( l=data.aa[,1], u=data.aa[,2],g.sc1= scale.X,g.sh1=shape.X,g.sc2=
    scale.Y,g.sh2=shape.Y)

  ll.aa      =  ifelse(ll.aa==0,max(ll.aa[ll.aa!=0]),ll.aa )

  lik.aa     =  sum(log(ll.aa))

  ll.crc     =  pc( l=data.crc[,1], u=data.crc[,2],g.sc1= scale.X,g.sh1=shape.X,g.
   sc2=scale.Y,g.sh2=shape.Y)

  ll.crc     =  ifelse(ll.crc==0,max(ll.crc[ll.crc!=0]),ll.crc)

  lik.crc    =  sum(log(ll.crc))


  log.lik    =  sum(lik.af,lik.aa,lik.crc)
  return(-log.lik)
}

#  Gompertz + Covariate and  Gompertz + Covariate
L6 = function(para, data=data.X) {

  data.af    =  subset(data,event==0,select=c(a,b))
  data.aa    =  subset(data,event==1,select=c(a,b))
  data.crc   =  subset(data,event==2,select=c(a,b))


  shape.X    =  as.matrix(rep(exp(para[1]),nrow(data)))            # Shape parameter
    for first transition X
  X          =  select(data,W)
  X1         =  data.matrix(cbind(1,X))
  beta.X     =  data.matrix(para[2:(ncol(X1)+1)])
  scale.X    =  exp(X1 %*%beta.X)                                    # Scale
   parameter for first transition X


  shape.Y    =  as.matrix(rep(exp(para[(ncol(X1)+2)]),nrow(data)))  # Shape
   parameter for second transition Y
  Y          =  select(data,W)
  Y1         =  data.matrix(cbind(1,Y))
  beta.Y     =  data.matrix(para[(ncol(X1)+3):( ncol(X1)+1 + ncol(Y1)+1  ) ])
  scale.Y    =  exp(Y1 %*%beta.Y)                                       # Scale
   parameter for second transition Y
```

```
  lik.af     =  sum(paf(g.sc1=scale.X[data$event==0,], g.sh1=shape.X[data$event
    ==0,], u=data.af[,2]))

  # The likelihood contribution for some AA and CRC individuals could be zero as a
    result of numeric  underflow that is
  # associated with the Gompertz distribution (due to the two exponent terms in the
     Gompertz distribution  formula for large values).
  # A quick and dirty trick is to replace such individuals with the max likelihood
    contribution value.

  ll.aa      =  pa( l=data.aa[,1], u=data.aa[,2],g.sc1= scale.X[data$event==1,],g.
    sh1=shape.X[data$event==1,],
  g.sc2= scale.Y[data$event==1,],g.sh2=shape.Y[data$event==1,])

  ll.aa      =  ifelse(ll.aa==0,max(ll.aa[ll.aa!=0]),ll.aa )

  lik.aa     =  sum(log(ll.aa))

  ll.crc     =  pc( l=data.crc[,1], u=data.crc[,2],g.sc1= scale.X[data$event==2,],g.
    sh1=shape.X[data$event==2,],
  g.sc2=scale.Y[data$event==2,],g.sh2=shape.Y[data$event==2,])

  ll.crc     =  ifelse(ll.crc==0,max(ll.crc[ll.crc!=0]),ll.crc)

  lik.crc    =  sum(log(ll.crc))




  log.lik   =  sum(c(lik.af,lik.aa,lik.crc))
  return(-log.lik)
}


# Loglogistic   and Loglogistic
L7 = function(para, data=data.X) {
  data.af    =  subset(data,event==0,select=c(a,b))
  data.aa    =  subset(data,event==1,select=c(a,b))
  data.crc   =  subset(data,event==2,select=c(a,b))
  scale.X    =  exp(para[1])
  shape.X    =  exp(para[2])
  scale.Y    =  exp(para[3])
  shape.Y    =  exp(para[4])
  lik.af     =  sum(paF(ll.sc1=scale.X,ll.sh1=shape.X, u=data.af[,2]))
  lik.aa     =  sum(log(pAa( l=data.aa[,1], u=data.aa[,2],ll.sc1= scale.X,ll.sh1=
    shape.X,ll.sc2= scale.Y,ll.sh2=shape.Y)))
  lik.crc    =  sum(log(pcRc( l=data.crc[,1], u=data.crc[,2],ll.sc1= scale.X,ll.sh1=
    shape.X,ll.sc2=scale.Y,ll.sh2=shape.Y)))
  log.lik    =  sum(lik.af,lik.aa,lik.crc)
  return(-log.lik)
}

# Lognormal  and Lognormal
L8 = function(para, data=data.X) {
  data.af    =  subset(data,event==0,select=c(a,b))
  data.aa    =  subset(data,event==1,select=c(a,b))
```

```
  data.crc   =  subset(data,event==2,select=c(a,b))
  meanlog.X =  para[1]   # This parameter is not defined in the log scale (i.e.
   using  exp()) since "mean" can be any real number
  sdlog.X    =  exp(para[2])
  meanlog.Y =  para[3]   # This parameter is not defined in the log scale (i.e.
   using exp()) since "mean" can be any real number
  sdlog.Y    =  exp(para[4])
  lik.af     =  sum(p.aF(sdlog1=sdlog.X,meanlog1=meanlog.X, u=data.af[,2]))
  lik.aa     =  sum(log(p.Aa( l=data.aa[,1], u=data.aa[,2],sdlog1= sdlog.X,meanlog1=
   meanlog.X,sdlog2= sdlog.Y,meanlog2=meanlog.Y)))
  lik.crc    =  sum(log(p.cRc ( l=data.crc[,1], u=data.crc[,2],sdlog1= sdlog.X,
   meanlog1=meanlog.X,sdlog2=sdlog.Y,meanlog2=meanlog.Y)))
  log.lik     = sum(lik.af,lik.aa,lik.crc)
  return(-log.lik)
}

# Gamma and Gamma
L9 = function(para, data=data.X) {
  data.af    =  subset(data,event==0,select=c(a,b))
  data.aa    =  subset(data,event==1,select=c(a,b))
  data.crc   =  subset(data,event==2,select=c(a,b))
  scale.X    =  exp(para[1])
  shape.X    =  exp(para[2])
  scale.Y    =  exp(para[3])
  shape.Y    =  exp(para[4])
  lik.af     =  sum(p.f(ga.sc1=scale.X,ga.sh1=shape.X, u=data.af[,2]))
  lik.aa     =  sum(log(p.a( l=data.aa[,1], u=data.aa[,2],ga.sc1= scale.X,ga.sh1=
   shape.X,ga.sc2= scale.Y,ga.sh2=shape.Y)))
  lik.crc    =  sum(log(p.c( l=data.crc[,1], u=data.crc[,2],ga.sc1= scale.X,ga.sh1=
   shape.X,ga.sc2=scale.Y,ga.sh2=shape.Y)))
  log.lik     = sum(lik.af,lik.aa,lik.crc)
  return(-log.lik)
}
```

4 maxmfun.R

```
library(numDeriv)
maxmfun2 = function(data.X,     # simulated data
p.X,         # covariate for first transition X? Two options: 1 = yes & 0 = No
p.Y,         # covariate for second transition Y? Two options: 1 = yes & 0 = No
init,        # initial values for the maximization
X.dist,      # Distribution of first transition X; options are Exp, Weibull,
   Gompertz, Loglogistic, Lognormal & Gamma
Y.dist       # Distribution of second transition Y; options are Exp, Weibull,
   Gompertz, Loglogistic, Lognormal & Gamma


){

  if(p.X==0&p.Y==0&X.dist=='Exp'&Y.dist=='Exp'){
    log.lik = L1
    nam = c('lambda1','lambda2', paste("se-",c('lambda1','lambda2'),sep=""))

  } else if(p.X==1&p.Y==1&X.dist=='Exp'&Y.dist=='Exp'){
    log.lik = L2
```

```
  nam  = c('alpha0','alpha1', 'beta0','beta1',paste("se-",c('alpha0','alpha1', '
 beta0','beta1'),sep=""))

} else if(p.X==0&p.Y==0&X.dist=='Weibull'&Y.dist=='Weibull'){
  log.lik = L3
  nam = c('scale.X','shape.X','scale.Y','shape.Y',paste("se-",c('scale.X','shape.
 X','scale.Y','shape.Y'),sep=""))
} else if(p.X==1&p.Y==1&X.dist=='Weibull'&Y.dist=='Weibull'){
  log.lik = L4
  nam =  c('shape.X','alpha0','alpha1', 'shape.Y', 'beta0','beta1',paste("se-",c(
 'shape.X','alpha0','alpha1', 'shape.Y', 'beta0','beta1'),sep=""))
}else if(p.X==0&p.Y==0&X.dist=='Gompertz'&Y.dist=='Gompertz'){
  log.lik = L5
  nam = c('scale.X','shape.X','scale.Y','shape.Y',paste("se-",c('scale.X','shape.
 X','scale.Y','shape.Y'),sep=""))
}else if(p.X==1&p.Y==1&X.dist=='Gompertz'&Y.dist=='Gompertz'){
  log.lik = L6
  nam =  c('shape.X','alpha0','alpha1', 'shape.Y', 'beta0','beta1',paste("se-",c(
 'shape.X','alpha0','alpha1', 'shape.Y', 'beta0','beta1'),sep=""))
} else if(p.X==0&p.Y==0&X.dist=='Loglogistic'&Y.dist=='Loglogistic'){
  log.lik = L7
  nam = c('scale.X','shape.X','scale.Y','shape.Y',paste("se-",c('scale.X','shape.
 X','scale.Y','shape.Y'),sep=""))
} else if(p.X==0&p.Y==0&X.dist=='Lognormal'&Y.dist=='Lognormal'){
  log.lik = L8
  nam = c('meanlog.X','sdlog.X','meanlog.Y','sdlog.X',paste("se-",c('meanlog.X','
 sdlog.X','meanlog.Y','sdlog.X'),sep=""))
} else if(p.X==0&p.Y==0&X.dist=='Gamma'&Y.dist=='Gamma'){
  log.lik = L9
  nam = c('scale.X','shape.X','scale.Y','shape.Y',paste("se-",c('scale.X','shape.
 X','scale.Y','shape.Y'),sep=""))
}
else{
  stop('Error: check model specifications')
}


# perform maximization using optim(). Note: the "control" arguments of optim()
 can be adjusted (see R help on optim () and Appendix E)


if((X.dist=='Exp'&Y.dist=='Exp'& p.X==0&p.Y==0)| (X.dist == 'Weibull' &  Y.dist
 == 'Weibull' & p.X==0&p.Y==0 ) |
(p.X==0&p.Y==0&X.dist=='Gompertz'&Y.dist=='Gompertz')| (p.X==0&p.Y==0&X.dist=='
 Loglogistic'&Y.dist=='Loglogistic')|
(p.X==0&p.Y==0&X.dist=='Gamma'&Y.dist=='Gamma')  ){
  opt= optim(init, fn=log.lik, method='Nelder-Mead', data.X, control = list(
 reltol=1e-16,maxit=5000))
  opt= optim(opt$par, fn=log.lik, method='BFGS',  gr = NULL,data.X, control =
 list(reltol=1e-16,maxit=5000))
  se= exp(opt$par)*sqrt(diag(solve(numDeriv::hessian(log.lik,opt$par))))
  est = round(c( exp(opt$par), se),3)

}else if(p.X==0&p.Y==0&X.dist=='Lognormal'&Y.dist=='Lognormal'){
  opt= optim(init, fn=log.lik, method='Nelder-Mead', data.X, control = list(
 reltol=1e-16,maxit=5000))
  opt= optim(opt$par, fn=log.lik, method='BFGS',  gr = NULL,data.X, control =
```

```
    list(reltol=1e-16,maxit=5000))
     SE = sqrt(diag(solve(numDeriv::hessian(log.lik,opt$par))))
     se= c( SE[1], exp(opt$par[2])*SE[2], SE[3], exp(opt$par[4])*SE[4] )

     est= c(opt$par[1], exp(opt$par[2]), opt$par[3], exp(opt$par[4]) , se)

     est = round(est,3)

  }else if(X.dist=='Exp'&Y.dist=='Exp'& p.X==1&p.Y==1){
     opt= optim(init, fn=log.lik, method='Nelder-Mead', data.X, control = list(
    reltol=1e-16,maxit=5000))
     opt= optim(opt$par, fn=log.lik, method='BFGS',  gr = NULL,data.X, control =
    list(reltol=1e-16,maxit=5000))
     se=sqrt(diag(solve(numDeriv::hessian(log.lik,opt$par))))
     est = round(c( opt$par, se),3)
  }
  else if((p.X==1&p.Y==1&X.dist=='Gompertz'&Y.dist=='Gompertz')| (X.dist == '
    Weibull' &  Y.dist == 'Weibull' & p.X==1&p.Y==1 )){
     opt= optim(init, fn=log.lik, method='Nelder-Mead', data.X, control = list(
    reltol=1e-16,maxit=5000))
     SE = sqrt(diag(solve(numDeriv::hessian(log.lik,opt$par))))
     se= c( exp(opt$par[1])*SE[1], SE[2:3], exp(opt$par[4])*SE[4], SE[5:6] )

     est= c(exp(opt$par[1]), opt$par[2:3], exp(opt$par[4]), opt$par[5:6], se)

     est = round(est,3)
  }

  else{
     stop('Error: check model specifications')
  }

  names(est) = nam
  return(est)
}
```

5 fxn.R

```
cal          = function(res.mat,  # matrix containing the Monte Carlo estimates
tval       # vector of true parameter values
){

  # calculate the proportion of successful iterations
  res.mat0   =  nrow(res.mat)
  res.mat    =  res.mat[complete.cases(res.mat),]
  q          =  100* (nrow(res.mat)/res.mat0 )


  # calculate the  Monte Carlo mean (MCM)
  p          =  ncol(res.mat)
  est.mat    =  res.mat[, 1:(p/2)]
  Est        =  colMeans(est.mat)

  # calculate the root mean square error (RMSE)
```

```
  bb         =  sweep(est.mat,2,tval)

  RMSE       =  sqrt(colMeans(bb^2))

  # calculate the Relative bias (RB)

  RB         =  100*((colMeans(est.mat)/tval)-1)

  # calculate the coefficient of variation (CV)
  CV         =  (apply(est.mat,2,sd))/tval


  # calculate the coverage rate (CR) and average width (AW)

  se.mat     =  res.mat[, (p/2 +1):p]
  se.mat0    =  2*qnorm(0.975)*se.mat
  ACIL       =  colMeans(se.mat0)

  CR <- rep(NA,ncol(se.mat))
  for(i in 1: ncol(se.mat)){
    lower<- est.mat[,i] -qnorm(0.975)*se.mat[,i]
    upper<- est.mat[,i]+qnorm(0.975)*se.mat[,i]

    int <- rep(NA,length(lower))
    for(ii in seq_along(lower)){
      int[ii] <-   ((lower[ii] < tval[i]) & (upper[ii] >tval[i]))
    }
    CR[i] <- mean(int)
  }

  # collate the performance measures
  perf.m <- data.frame(True= round(tval,2), MCM=round(Est,2), RMSE=round(RMSE,3),
   RB=round(RB,1),
  CV=round(CV,3), CR=round(CR,3), AW=round(ACIL,2), Percentage.sucess=q)

  return(perf.m)
}
```

6 simDemo1.R

```
# clear R
rm(list = ls())

# load required packages
library(tidyverse)
library(numDeriv)

# load required functions
source('simData.R')
source('probExpressions.R')
source('likelihood.R')
source('maxmfun.R')

# set.seed(2020)     # set seed for reproducibility
```

```
start_time =  proc.time()   # Start timing
# First check the required arguments for the dat2() function before use.
data.X   =   dat(n=5000,       # Number of individuals; the larger the sample size,
   the better the estimates
X.dist  = 'Weibull',   # Distribution of first transition X; options are Exp,
   Weibull , Gompertz, Loglogistic, Lognormal & Gamma
Y.dist  = 'Weibull',   # Distribution of second transition Y; options are Exp,
   Weibull ,Gompertz, Loglogistic, Lognormal & Gamma
alpha0  = NULL,   # Regression intercept for first transition X
alpha1  = NULL,   # Regression coefficient of covariate W for first transition X
beta0   = NULL,   # Regression intercept for second transition Y
beta1   = NULL,   # Regression coefficient of covariate W for second transition Y
X.rate  = NULL,   # Exponential rate parameter for first transition X
Y.rate  = NULL,   # Exponential rate parameter for second transition Y
X.scale = 15,    # Weibull, Gompertz,  Loglogistic & Gamma scale parameter for
   first transition X
X.shape = 1.5,    # Weibull, Gompertz,  Loglogistic & Gamma shape parameter for
   first transition X
Y.scale = 5.0,    # Weibull, Gompertz,  Loglogistic & Gamma scale parameter for
   second transition Y
Y.shape = 0.8,    # Weibull, Gompertz,  Loglogistic & Gamma shape parameter  for
   second transition Y
X.meanlog   =    NULL,  # Lognormal mean parameter for first transition X
X.sdlog     =    NULL,  # Lognormal standard deviation parameter for first
   transition X
Y.meanlog   =    NULL,  # Lognormal mean parameter for second transition Y
Y.sdlog     =    NULL,  # Lognormal standard deviation parameter for second
   transition Y
p.X    = 0,            # covariate for first transition X? Two options: 1 = yes & 0
    = No
p.Y    = 0             # covariate for second transition Y? Two options: 1 = yes & 0
    = No

)

# print random 10 rows of the data.
# (a, b) are the interval-censored data, event = health state (0=AF, 1 = AA, 2= CRC
   ),
# w = standard normal distributed covariate
print(dplyr::sample_n(data.X , 10) )

# Compute the proportion of health states
# Note that the sample size n and the proportion of health states (particularly for
    CRC) strongly affects the estimates.
print(100*prop.table(table(data.X$event)) )

# First check the required arguments for the maxmfun2() function before use.
result = maxmfun2(data.X   =  data.X, # simulated data
p.X      =  0,      # covariate for first transition X? Two options: 1 = yes & 0 =
   No
p.Y      =  0,      # covariate for second transition Y? Two options: 1 = yes & 0 =
   No
init     =  c(-0.1, -0.1, -0.1, -0.1),   # initial values for the maximization
X.dist   =   'Weibull',     # Distribution of first transition X; options are Exp,
   Weibull, Gompertz, Loglogistic, Lognormal & Gamma
Y.dist   =   'Weibull'      # Distribution of second transition Y; options are Exp,
```

```
    Weibull, Gompertz, Loglogistic, Lognormal & Gamma

)
print(proc.time() - start_time)  # calculate computational time

# output estimates (in log scale for rate, scale, shape and sdlog parameters)
# Use exp() to return to estimates in original scale if needed

print(result)
```

7 simDemo2.R

```
# clear R
rm(list = ls())

# load required packages
library(tidyverse)
library(numDeriv)

# load required functions
source('simData.R')
source('probExpressions.R')
source('likelihood.R')
source('maxmfun.R')
source('fxn.R')

# set.seed(2020)      # set seed for reproducibility

start_time =  proc.time()   # Start timing
nsim       =  100          # Number of simulation
p          =  4            # Total number of true parameter values


res        =  matrix(NA,nrow = nsim,ncol = 2*p,byrow = TRUE)

# Start Monte Carlo simulation

for(ii in 1:nsim){
  tryCatch(
  expr  = {
    # check the required arguments for the dat() function by first reading the
    sumData.R file
    data.X   =   dat(n=5000,        # Number of individuals; the larger the sample
    size, the better the estimates
    X.dist   = 'Weibull',   # Distribution of first transition X; options are Exp,
    Weibull , Gompertz, Loglogistic, Lognormal & Gamma
    Y.dist   = 'Weibull',   # Distribution of second transition Y; options are Exp,
    Weibull ,Gompertz, Loglogistic, Lognormal & Gamma
    alpha0   = NULL,    # Regression intercept for first transition X
    alpha1   = NULL,    # Regression coefficient of covariate W for first transition
    X
    beta0    = NULL,    # Regression intercept for second transition Y
    beta1    = NULL,    # Regression coefficient of covariate W for second transition
    Y
    X.rate   = NULL,    # Exponential rate parameter for first transition X
```

```
    Y.rate   = NULL,   # Exponential rate parameter for second transition Y
    X.scale = 15,    # Weibull, Gompertz,  Loglogistic & Gamma scale parameter for
   first transition X
    X.shape = 1.5,    # Weibull, Gompertz,  Loglogistic & Gamma shape parameter for
    first transition X
    Y.scale = 5.0,    # Weibull, Gompertz,  Loglogistic & Gamma scale parameter for
    second transition Y
    Y.shape = 0.8,    # Weibull, Gompertz,  Loglogistic & Gamma shape parameter
   for second transition Y
    X.meanlog   =    NULL,  # Lognormal mean parameter for first transition X
    X.sdlog     =    NULL,  # Lognormal standard deviation parameter for first
   transition X
    Y.meanlog   =    NULL,  # Lognormal mean parameter for second transition Y
    Y.sdlog     =    NULL,  # Lognormal standard deviation parameter for second
   transition Y
    p.X    = 0,            # covariate for first transition X? Two options: 1 = yes
    & 0 = No
    p.Y    = 0            # covariate for second transition Y? Two options: 1 = yes
    & 0 = No

    )
    # First check the required arguments for the maxmfun2() function before use.
    res[ii,] = maxmfun2(data.X  =  data.X, # simulated data
    p.X     =  0,      # covariate for first transition X? Two options: 1 = yes & 0
    = No
    p.Y     =  0,      # covariate for second transition Y? Two options: 1 = yes &
   0 = No
    init    =  c(-0.1, -0.1, -0.1, -0.1),   # initial values for the maximization
    X.dist  =   'Weibull',     # Distribution of first transition X; options are
   Exp, Weibull, Gompertz, Loglogistic, Lognormal & Gamma
    Y.dist  =   'Weibull'      # Distribution of second transition Y; options are
   Exp, Weibull, Gompertz, Loglogistic, Lognormal & Gamma

    )

  },
  error = function(e){
    return(NA)


  },
  warning = function(w){
    return(NA)
  }
  )



}

print(proc.time() - start_time)  # calculate computational time
# print(res)



# Obtain the performance measures. First check the arguments of the cal() function
   by reading the fxn.R file
```

```
result <- cal(res,c( 15, 1.5, 5.0, 0.8))  # supply  true parameter values as second
    argument
#
print(result)
```

References

[1] Collett D. *Modelling survival data in medical research.* CRC press; 2015.
