# Supplementary material for: A progressive three-state model to estimate time to cancer: a likelihood-based approach
Source: BMC Med Res Methodol. 2022 Jun 27;22:179. doi: 10.1186/s12874-022-01645-2 (PMC9235269; doi:10.1186/s12874-022-01645-2)
Supplement: Supplementary file 2 — Additional file 2 Supplementary Material. [file 12874_2022_1645_MOESM2_ESM.pdf]

Additional file 2: Supplementary Material

Introduction

We present detailed information and additional results that are not presented in the main manuscript. The reader is referred to the main manuscript for all terms and symbols not explicitly defined here This document is organized as follows:

A Detailed derivation of the joint distribution for transition times (X, Y) for any assumed probability distribution **2**

B Model with time-invariant covariates **3**

C Detailed derivation of the proposed model specifications without covariates when X and Y are both exponentially distributed **4**

D Implementation details **6**

E Detailed data simulation procedure **7**

F Additional simulation results **8**

G Comparing true survival curves with proposed method and the 2-phase semi-Markov model **10**

H Results for the Norwegian data analysis **11**

A Detailed derivation of the joint distribution for transition times (X, Y) for any assumed probability distribution

The derivations below are for the three probability expressions reported in the *Model* section of the main manuscript.

Let the density and distribution functions of X and Y be f, F; and g, G, respectively. For

1. no event until last follow-up time visit v_m.

$$\begin{aligned}\Pr\Big(v_{m-1} < v_m < X < Z\Big) &= \Pr\Big(X > v_m\Big),\\ &= 1 - \Pr\Big(X \leq v_m\Big),\\ &= 1 - F(v_m).\end{aligned} \tag{1}$$

2. AA between v_{m-1} and v_m but no CRC before v_m.

$$\begin{aligned}\Pr\Big(v_{m-1} < X < v_m; Z > v_m\Big) &= \Pr\Big(v_{m-1} < X < v_m; X + Y > v_m\Big),\\ &= \Pr\Big(v_{m-1} < X < v_m; Y > v_m - X\Big),\\ &= \int_{v_{m-1}}^{v_m} \int_{v_m - x}^{\infty} f(x) g(y|x) dy dx,\\ &= \int_{v_{m-1}}^{v_m} f(x) \int_{v_m - x}^{\infty} g(y) dy dx,\\ &= \int_{v_{m-1}}^{v_m} f(x) \Big[1 - G(v_m - x)\Big] dx,\\ &= \int_{v_{m-1}}^{v_m} f(x) dx - \int_{v_{m-1}}^{v_m} f(x) G(v_m - x) dx,\\ &= F(v_m) - F(v_{m-1}) - \int_{v_{m-1}}^{v_m} f(x) G(v_m - x) dx.\end{aligned} \tag{2}$$

3. CRC between v_{m-1} and v_m but no AA before v_{m-1}.

$$\begin{aligned}\Pr\Big(v_{m-1} < X < v_m; v_{m-1} < Z < v_m\Big) &= \Pr\Big(v_{m-1} < X < v_m; v_{m-1} < X + Y < v_m\Big),\\ &= \Pr\Big(v_{m-1} < X < v_m; v_{m-1} - X < Y < v_m - X\Big),\\ &= \Pr\Big(v_{m-1} < X < v_m; 0 < Y < v_m - X\Big),\\ &= \int_{v_{m-1}}^{v_m} \int_{0}^{v_m - x} f(x) g(y|x) dy dx,\\ &= \int_{v_{m-1}}^{v_m} f(x) \int_{0}^{v_m - x} g(y) dy dx,\\ &= \int_{v_{m-1}}^{v_m} f(x) G(v_m - x) dx.\end{aligned} \tag{3}$$

B Model with time-invariant covariates

In this section, we will describe how certain patient characteristics or risk factors such as sex and age may affect the hazard function for the durations X and/or Y. These covariates are *time-invariant* if they do not change with time. The proportional hazards (PH) model is the most commonly used approach in examining this effect when the baseline hazard function is assumed to follow a specific probability distribution. The PH model is written as

$$h(t_i|\boldsymbol{w}^{(i)}) = h_0(t_i)\exp\left(\boldsymbol{\beta}'\boldsymbol{w}^{(i)}\right) \tag{4}$$

where $\boldsymbol{w}^{(i)} = \left(w_1^{(i)}, w_2^{(i)}, \cdots, w_p^{(i)}\right)'$ is a $p \times 1$ vector of covariates, and $\boldsymbol{\beta} = (\beta_1, \beta_2, \cdots, \beta_p)'$ is the corresponding $p \times 1$ vector of regression parameters of $\boldsymbol{w}^{(i)}$ for individual i, and $h_0(t_i)$ is the baseline hazard function at time t_i.

To examine the effect of $\boldsymbol{w}^{(i)}$ on the hazard when $h_0(t_i)$ is assumed to be exponentially distributed, we simply replace $h_0(t_i)$ in Equation (4) with the corresponding hazard function of an exponential distribution, that is, $h(t) = \lambda$ to obtain a new hazard function given by

$$h(t_i|\boldsymbol{w}^{(i)}) = \lambda\exp\left(\boldsymbol{\beta}'\boldsymbol{w}^{(i)}\right). \tag{5}$$

From Equation (5), we can see that survival time T_i is still exponentially distributed with new rate parameter $\lambda\exp\left(\boldsymbol{\beta}'\boldsymbol{w}^{(i)}\right)$; since an exponential rate parameter is equivalent to its hazard function.

Similarly, if $h_0(t_i)$ is assumed to be Weibull distributed, we simply replace $h_0(t_i)$ in Equation (4) with its hazard function $\frac{k}{\theta}\left(\frac{t}{\theta}\right)^{\kappa-1}$ to obtain a new hazard function given by

$$h(t_i|\boldsymbol{w}^{(i)}) = \frac{\kappa}{\theta}\left(\frac{t_i}{\theta}\right)^{\kappa-1}\exp\left(\boldsymbol{\beta}'\boldsymbol{w}^{(i)}\right). \tag{6}$$

From Equation (6), it can easily be shown that survival time T_i is still Weibull distributed with shape parameter κ but with a new scale parameter $\theta\exp\left(-\boldsymbol{\beta}'\boldsymbol{w}^{(i)}/\kappa\right)$.

C Detailed derivation of the proposed model specifications without covariates when X and Y are both exponentially distributed

Let $f(x) = \lambda_1 e^{-\lambda_1 x}$, $F(x) = 1 - e^{-\lambda_1 x}$, and $G(y) = 1 - e^{-\lambda_2 y}$.

1. From Equation (1), we have that:

$$\begin{aligned} \Pr\left(X > v_m \middle| \lambda_1\right) &= 1 - F(v_m), \\ &= 1 - (1 - e^{-\lambda_1 v_m}) = e^{-\lambda_1 v_m}. \end{aligned} \tag{7}$$

2. From Equation (2), we have that:

$$\begin{aligned} \Pr\left(v_{m-1} < X < v_m, Z > v_m \middle| \lambda_1, \lambda_2\right) &= \int_{v_{m-1}}^{v_m} f(x)\left[1 - G(v_m - x)\right] dx, \\ &= \int_{v_{m-1}}^{v_m} \lambda_1 e^{-\lambda_1 x} \times e^{-\lambda_2 (v_m - x)} dx, \\ &= \lambda_1 \int_{v_{m-1}}^{v_m} e^{-(\lambda_1 - \lambda_2)x - \lambda_2 v_m} dx, \\ &= \frac{\lambda_1}{-(\lambda_1 - \lambda_2)} \left[e^{-(\lambda_1 - \lambda_2)x - \lambda_2 v_m} \right]_{v_{m-1}}^{v_m}, \\ &= \frac{\lambda_1}{(\lambda_2 - \lambda_1)} \left[e^{-(\lambda_1 - \lambda_2)v_m - \lambda_2 v_m} - e^{-(\lambda_1 - \lambda_2)v_{m-1} - \lambda_2 v_m} \right], \\ &= \frac{\lambda_1}{(\lambda_2 - \lambda_1)} \left[e^{-\lambda_1 v_m} - e^{-\lambda_2 (v_m - v_{m-1}) - \lambda_1 v_{m-1}} \right], \quad \lambda_1 \neq \lambda_2. \end{aligned} \tag{8}$$

Similarly, when $\lambda = \lambda_1 = \lambda_2$, we have

$$\begin{aligned} \Pr\left(v_{m-1} < X < v_m, Z > v_m \middle| \lambda\right) &= \int_{v_{m-1}}^{v_m} f(x)\left[1 - G(v_m - x)\right] dx, \\ &= \int_{v_{m-1}}^{v_m} \lambda e^{-\lambda x} \times e^{-\lambda (v_m - x)} dx, \\ &= \lambda \int_{v_{m-1}}^{v_m} e^{-\lambda x - \lambda v_m + \lambda x} dx \\ &= \lambda \int_{v_{m-1}}^{v_m} e^{-\lambda r} dx, \\ \lambda e^{-\lambda v_m} & \int_{v_{m-1}}^{v_m} dx, \\ &= \lambda e^{-\lambda v_m} \left(v_m - v_{m-1}\right), \\ &= \lambda \left(v_m - v_{m-1}\right) e^{-\lambda v_m}. \end{aligned} \tag{9}$$

3. From Equation (3), we have that:

$$
\begin{aligned}
\Pr\left(v_{m-1} < X < v_m, v_{m-1} < Z < v_m \middle| \lambda_1, \lambda_2\right) &= \int_{v_{m-1}}^{v_m} f(x)G(v_m - x)dx, \\
&= \int_{v_{m-1}}^{v_m} \lambda_1 e^{-\lambda_1 x} \times \left(1 - e^{-\lambda_2 (v_m - x)}\right) dx, \\
&= \int_{v_{m-1}}^{v_m} \lambda_1 e^{-\lambda_1 x} dx - \int_{v_{m-1}}^{v_m} \lambda_1 e^{-\lambda_1 x} \times e^{-\lambda_2 (v_m - x)} dx, \\
&= \Bigg[- e^{-\lambda_1 x} \Bigg]_{v_{m-1}}^{v_m} - \int_{v_{m-1}}^{v_m} \lambda_1 e^{-\lambda_1 x} \times e^{-\lambda_2 (v_m - x)} dx, \\
&= e^{-\lambda_1 v_{m-1}} - e^{-\lambda_1 v_m} - \frac{\lambda_1}{(\lambda_2 - \lambda_1)} \Bigg[e^{-\lambda_1 v_m} - e^{-\lambda_2 (v_m - v_{m-1}) - \lambda_1 v_{m-1}} \Bigg] \quad \text{(see proof number 2)}, \\
&= e^{-\lambda_1 v_{m-1}} - e^{-\lambda_1 v_m} - \frac{\lambda_1}{(\lambda_2 - \lambda_1)} e^{-\lambda_1 v_m} + \frac{\lambda_1}{(\lambda_2 - \lambda_1)} e^{-\lambda_2 (v_m - v_{m-1}) - \lambda_1 v_{m-1}}, \\
&= e^{-\lambda_1 v_{m-1}} \Bigg[1 - e^{-\lambda_1 (v_m - v_{m-1})} - \frac{\lambda_1}{(\lambda_2 - \lambda_1)} e^{-\lambda_1 (v_m - v_{m-1})} + \frac{\lambda_1}{(\lambda_2 - \lambda_1)} e^{-\lambda_2 (v_m - v_{m-1}) - \lambda_1 v_{m-1} + \lambda_1 v_{m-1}} \Bigg], \\
&= e^{-\lambda_1 v_{m-1}} \Bigg[1 - e^{-\lambda_1 (v_m - v_{m-1})} - \frac{\lambda_1}{(\lambda_2 - \lambda_1)} e^{-\lambda_1 (v_m - v_{m-1})} + \frac{\lambda_1}{(\lambda_2 - \lambda_1)} e^{-\lambda_2 (v_m - v_{m-1})} \Bigg], \\
&= e^{-\lambda_1 v_{m-1}} \Bigg[1 - \frac{e^{-\lambda_1 (v_m - v_{m-1})} (\lambda_2 - \lambda_1) - \lambda_1 e^{-\lambda_1 (v_m - v_{m-1})}}{(\lambda_2 - \lambda_1)} + \frac{\lambda_1}{(\lambda_2 - \lambda_1)} e^{-\lambda_2 (v_m - v_{m-1})} \Bigg] \\
&= e^{-\lambda_1 v_{m-1}} \Bigg[1 - \frac{\lambda_2 e^{-\lambda_1 (v_m - v_{m-1})} + \lambda_1 e^{-\lambda_1 (v_m - v_{m-1})} - \lambda_1 e^{-\lambda_1 (v_m - v_{m-1})}}{(\lambda_2 - \lambda_1)} + \frac{\lambda_1}{(\lambda_2 - \lambda_1)} e^{-\lambda_2 (v_m - v_{m-1})} \Bigg], \\
&= e^{-\lambda_1 v_{m-1}} \Bigg[1 - \frac{\lambda_2}{(\lambda_2 - \lambda_1)} e^{-\lambda_1 (v_m - v_{m-1})} + \frac{\lambda_1}{(\lambda_2 - \lambda_1)} e^{-\lambda_2 (v_m - v_{m-1})} \Bigg], \quad \lambda_1 \neq \lambda_2.
\end{aligned} \tag{10}
$$

Similarly, when $\lambda = \lambda_1 = \lambda_2$, we have

$$
\begin{aligned}
\Pr\left(v_{m-1} < X < v_m, v_{m-1} < Z < v_m \middle| \lambda\right) &= \int_{v_{m-1}}^{v_m} f(x)G(v_m - x)dx, \\
&= \int_{v_{m-1}}^{v_m} \lambda e^{-\lambda x} \times \left(1 - e^{-\lambda (v_m - x)}\right) dx, \\
&= \int_{v_{m-1}}^{v_m} \lambda e^{-\lambda x} dx - \int_{v_{m-1}}^{v_m} \lambda e^{-\lambda x} \times e^{-\lambda (v_m - x)} dx, \\
&= \Bigg[- e^{-\lambda x} \Bigg]_{v_{m-1}}^{v_m} - \int_{v_{m-1}}^{v_m} \lambda e^{-\lambda x} \times e^{-\lambda (v_m - x)} dx, \\
&= \Bigg[- e^{-\lambda x} \Bigg]_{v_{m-1}}^{v_m} - \lambda (v_m - v_{m-1}) e^{-\lambda v_m} \quad \text{(see proof number 2)}, \\
&= e^{-\lambda v_{m-1}} - e^{-\lambda v_m} - \lambda (v_m - v_{m-1}) e^{-\lambda v_m}, \\
&= e^{-\lambda v_{m-1}} - e^{-\lambda v_m} \left[1 + \lambda (v_m - v_{m-1}) \right].
\end{aligned} \tag{11}
$$

D Implementation details

Two optimization methods in `optim()` function are used to maximize the joint log-likelihood function reported in the *Likelihood Construction and Estimation* section of the main manuscript. First, the `Nelder-Mead` method [1], and then the quasi–Newton `BFGS` (Broyden–Fletcher–Goldfarb–Shanno) method [2] afterwards. With sensible initial values, the `par` outputs from the `Nelder-Mead` method serve as good starting values for the `BFGS` method. The asymptotic standard errors (SEs) are computed from the Hessian matrix obtained using the `hessian()` function in the `numDeriv` package in **R**, which implements the Richardson's extrapolation method of numerical differentiation; a well known method for improving the accuracy of approximations [3]. During the optimization process, for models without covariates, the exponential rate, Weibull scale and shape parameters, respectively, are defined on the log scale (i.e., `exp()`). This is to ensure that they are always positive in the parameter space. Their respective ML estimates and SEs can be are obtained by transforming back to the original scale of the parameters. A finite-difference approximation (default) is used if analytical derivatives are not supplied. To improve convergence, the optimization settings in `optim()` could be adjusted. For example, the `maxit` argument can be increased but this is not a guarantee. Likelihood function involving probability expressions reported in Appendix A are approximated by numerical integration using the `integrate()` function in base **R**, which implements the adaptive quadrature rule over the integration interval $(\upsilon_{m-1}, \upsilon_m)$. Numerical problems while using `optim()` or `integrate()` functions could be encountered. For instance, when poor choice of initial values are used for `optim()` or divergent issues when performing numerical integration. The divergent issues could happen when the integrand is infinite at some point(s) within the integration interval $(\upsilon_{m-1}, \upsilon_m)$. This can sometimes be resolved by either lowering the default tolerance (=0.0001220703) in the `integrate()` function using the `rel.tol` argument, or by scaling the input variables to avoid numeric overflow or underflow encountered when taking the exponent of values of large magnitude during the numerical integration process. Also, parameter identifiability issues or failure of the Hessian matrix to be positive definite could be encountered when insufficient data are used to fit models with too many parameters [4]. Hence, it is recommended to use simpler models if there are not enough data. When the so-called false convergence, a situation where the optimization algorithm reports a solution (i.e., convergence) but the Hessian matrix which is needed for computing the SE of the model parameters fails to be positive definite [5], is encountered, the default relative convergence tolerance (about 1e−8) in `optim()` could be lower using its `rel.tol` argument. Another possible solution is to use only the `Nelder-Mead` method in `optim()` while performing the optimization, particularly for likelihood functions that are difficult to differentiate. Finally, since there are no direct procedures to compute the 95% confidence interval (CI) for the estimate of the median transition times, a parametric bootstrap procedure as recommend by Rice [6] can be used, if the median transition times are sought. For example, to compute the 95% CI for the median estimate for X with cdf F, we proceed as follows:

(i) Compute the median estimate, say $\widetilde{\psi}$ of X using the ML estimate of X as input parameter(s) for F.

(ii) Draw J samples x_j^*, $j = 1, 2, \cdots, J$ from F.

(iii) Obtain B bootstrap estimates of the median $\widetilde{\psi}_b^*$ from the J draws in (ii), where $b = 1, 2, \cdots, B$.

(iv) For each median obtained in (iii), compute the bootstrap difference $\delta_b^* = \widetilde{\psi}_b^* - \widetilde{\psi}$.

(v) Compute the 0.05 and 0.95 quantile for δ_b^* as δ_1^* and δ_2^*, respectively.

(vi) The 95% CI for the median estimate $\widetilde{\psi}$ is then obtained as $\left(\widetilde{\psi} - \delta_2^*, \widetilde{\psi} - \delta_1^*\right)$

E Detailed data simulation procedure

The following steps were used to simulate a realistic though hypothetical surveillance study with w as a continuous covariate:

Step 1: Create a hypothetical cohort of n individuals who enter the surveillance after complete removal of their adenomas via colonoscopic polypectomy. Choose a model specification for X and Y. Proceed to **Step 3** if the chosen model does not depend on covariate. Else proceed to the next step.

Step 2: For models that are covariate-dependent, generate one covariate for $i = 1, 2, \cdots, n$ number of individuals

$$w^{(i)} \sim N(0, 1).$$

Step 3: For models that are covariate-dependent, set the corresponding regression parameter values for X and/or Y. That is, (α_0, α_1) and/or (β_0, β_1) as regression intercept and regression coefficient of w for X and/or Y, respectively. If X and/or Y are independent of the covariate, choose parameter values for the distributions of X and/or Y.

Step 4: Generate the transition times X and Y from the respective chosen probability distributions for $i = 1, 2, \cdots, n$ individuals. For example, if

(i) X is exponentially distributed with rate parameter λ_1 and is independent of w as covariate, then $X^{(i)} \sim Exp(\lambda_1)$.

(ii) X is exponentially distributed with rate parameter λ_1 given w as covariate, then $X^{(i)} \sim Exp(\lambda_1^{(i)}, w^{(i)})$ where $\lambda_1^{(i)} = \exp(\alpha_0 + \alpha_1 \times w^{(i)})$.

(iii) X is Weibull distributed with scale and shape parameters θ_1, κ_1, respectively, and is independent of w as covariate, then $X^{(i)} \sim Weibull\,(\theta_1, \kappa_1)$.

(iv) X is Weibull distributed with scale and shape parameters θ_1, κ_1, respectively, given w as covariate, then $X^{(i)} \sim Weibull\left(\theta_1^{(i)}, \kappa_1, w^{(i)}\right)$ where $\theta_1^{(i)} = \exp\left\{\alpha_0 - \left(\alpha_1 \times w^{(i)}\right)/\kappa_1\right\}$.

A similar approach is employed for Y. Since in reality we cannot observe the time Y, but only the times X and $Z = X + Y$, we summed the simulated durations X and Y to obtain the time Z for each individual. Therefore, for our simulation study and consequently for our proposed models, we only need the observed durations X and Z for each individual.

Step 5: For each individual i, generate a vector $\boldsymbol{v}^{(i)}$ of follow-up visits independent of X and Y from a uniform distribution over $[a, b]$, where a and b are the minimum and maximum years of follow-up of the entire cohort, respectively.

Step 6: For each individual i, compare the observed times $X^{(i)}$ and $Z^{(i)}$ generated in **Step 4** with the individual's vector of follow-up visit times $\boldsymbol{v}^{(i)}$ generated in **Step 5**. Register the follow-up visit at which either AA or CRC is present, as well as the timing of the last visit before that. For individuals who had no AA or CRC throughout their follow-up period, register their last visit.

F Additional simulation results

This section contains the results of additional simulation study under Scenario II, referenced in the *Results* section of the main manuscript in order to demonstrate the effect of increasing the proportion of CRCs in the sample.

Table S1 Results of 1000 MC Simulations for Models Under on Scenario II

			$n = 1000$						$n = 5000$					
Model	PAR	True	MCM	RMSE	RB [a]	CV	CR	AW	MCM	RMSE	RB[a]	CV	CR	AW
M1	λ_1	0.07	0.08	0.003	0.0	0.04	0.952	0.01	0.07	0.001	0.0	0.02	0.964	0.00
	λ_2	0.20	0.20	0.014	0.0	0.07	0.947	0.05	0.20	0.006	0.0	0.03	0.951	0.02
M2	α_0	−2.60	−2.60	0.038	0.0	−0.01	0.950	0.15	−2.60	0.017	0.0	−0.01	0.945	0.07
	α_1	0.05	0.05	0.038	−1.3	0.76	0.956	0.15	0.05	0.017	−0.8	0.34	0.951	0.07
	λ_2	0.20	0.20	0.014	0.1	0.07	0.953	0.05	0.20	0.006	0.1	0.03	0.946	0.02
M3	λ_1	0.08	0.08	0.003	0.1	0.04	0.950	0.01	0.08	0.001	0.0	0.02	0.954	0.00
	β_0	−2.10	−2.10	0.123	0.1	−0.06	0.955	0.50	−2.10	0.056	0.2	−0.02	0.945	0.22
	β_1	−3.00	−3.02	0.218	0.8	−0.07	0.960	0.86	−3.01	0.094	0.3	−0.03	0.962	0.38
M4	α_0	−1.10	−1.09	0.068	−0.5	−0.06	0.948	0.26	−1.10	0.030	−0.2	−0.03	0.948	0.12
	α_1	−3.00	−3.01	0.117	0.3	−0.04	0.954	0.47	−3.00	0.053	0.1	−0.02	0.959	0.21
	β_0	−2.30	−2.31	0.100	0.2	−0.04	0.946	0.38	−2.30	0.043	0.1	−0.02	0.951	0.17
	β_1	−1.00	−1.01	0.096	0.7	−0.10	0.952	0.38	−1.00	0.042	0.2	−0.04	0.954	0.17
M5	λ_1	0.08	0.08	0.003	0.1	0.04	0.953	0.01	0.08	0.001	0.0	0.02	0.951	0.01
	κ_2	2.00	2.05	0.332	2.6	0.16	0.948	1.24	2.01	0.142	0.1	0.07	0.946	0.53
	θ_2	4.00	4.02	0.230	0.4	0.06	0.945	0.89	4.00	0.103	0.1	0.03	0.943	0.39
M6	α_0	−0.50	−0.51	0.083	1.5	−0.17	0.942	0.34	−0.50	0.038	0.5	−0.07	0.950	0.15
	α_1	−4.00	−3.99	0.156	−0.4	−0.04	0.926	0.64	−4.00	0.073	−0.1	−0.02	0.940	0.30
	κ_2	2.50	2.53	0.213	1.2	0.08	0.949	0.80	2.50	0.089	0.1	0.04	0.959	0.35
	θ_2	4.50	4.50	0.151	−0.1	0.03	0.946	0.57	4.50	0.064	−0.1	0.01	0.952	0.26
M7	λ_1	0.08	0.08	0.003	0.2	0.04	0.955	0.01	0.08	0.001	0.0	0.02	0.950	0.01
	κ_2	0.50	0.51	0.115	1.3	0.23	0.954	0.45	0.50	0.050	0.3	0.10	0.949	0.20
	β_0	2.50	2.57	0.430	2.9	0.17	0.935	1.60	2.51	0.168	0.6	0.07	0.957	0.65
	β_1	1.00	1.01	0.083	1.0	0.08	0.968	0.33	1.00	0.037	0.3	0.04	0.953	0.15
M8	α_0	−2.50	−2.50	0.040	0.0	−0.02	0.954	0.16	−2.50	0.018	0.0	−0.01	0.964	0.07
	α_1	−0.80	−0.80	0.042	0.4	−0.05	0.954	0.17	−0.80	0.019	0.0	−0.02	0.956	0.07
	κ_2	0.80	0.80	0.134	0.2	0.17	0.954	0.51	0.80	0.057	0.0	0.07	0.955	0.23
	β_0	1.50	1.51	0.125	0.7	0.08	0.952	0.47	1.50	0.050	0.2	0.03	0.963	0.20
	β_1	0.80	0.81	0.093	0.8	0.12	0.955	0.36	0.80	0.042	0.1	0.05	0.938	0.16
M9	κ_1	0.18	0.18	0.016	0.9	0.09	0.944	0.06	0.18	0.008	0.1	0.04	0.941	0.03
	θ_1	5.00	5.13	1.163	2.5	0.23	0.947	4.43	5.01	0.507	0.2	0.10	0.942	1.95
	λ_2	0.18	0.18	0.011	0.3	0.06	0.950	0.04	0.18	0.005	−0.1	0.03	0.950	0.02

(continued)

Table S1 (continued)

Model	PAR	True	$n = 1000$						$n = 5000$					
			MCM	RMSE	RB[a]	CV	CR	AW	MCM	RMSE	RB[a]	CV	CR	AW
M10	κ_1	0.20	0.20	0.018	0.3	0.09	0.941	0.07	0.20	0.008	0.0	0.04	0.949	0.03
	α_0	1.50	1.49	0.212	−0.6	0.14	0.952	0.83	1.50	0.095	0.0	0.06	0.945	0.37
	α_1	0.50	0.50	0.041	0.0	0.08	0.950	0.16	0.50	0.018	0.3	0.04	0.959	0.07
	λ_2	0.16	0.16	0.010	0.3	0.06	0.947	0.04	0.16	0.005	0.0	0.03	0.958	0.02
M13	κ_1	1.50	1.50	0.059	0.0	0.04	0.944	0.23	1.50	0.026	0.0	0.02	0.955	0.10
	θ_1	15.00	15.01	0.394	0.0	0.03	0.949	1.53	15.01	0.165	0.0	0.01	0.955	0.68
	κ_2	0.80	0.81	0.138	1.7	0.17	0.953	0.54	0.81	0.062	0.7	0.08	0.950	0.24
	θ_2	5.00	5.09	0.687	1.7	0.14	0.930	2.55	5.01	0.275	0.2	0.06	0.945	1.05
M14	κ_1	0.20	0.20	0.018	0.3	0.09	0.950	0.07	0.20	0.008	0.1	0.04	0.951	0.03
	α_0	1.50	1.51	0.207	0.4	0.14	0.956	0.81	1.50	0.093	0.1	0.06	0.947	0.36
	α_1	0.50	0.50	0.041	0.5	0.08	0.958	0.16	0.50	0.019	0.0	0.04	0.951	0.07
	κ_2	2.50	2.53	0.240	1.2	0.10	0.960	0.93	2.51	0.106	0.3	0.04	0.945	0.41
	θ_2	4.50	4.50	0.169	0.0	0.04	0.949	0.66	4.50	0.073	0.1	0.02	0.962	0.30

PAR, parameter; MCM, Monte Carlo means; RMSE, root mean squared error; RB, relative bias % ; CV, coefficient of variation; AW, average confidence interval width.

[a] The negative signs correspond to underestimation (overestimation) for positive (negative) true values while the positive signs correspond to overestimation (underestimation) for positive (negative) true values. The 0.0 values are due to approximation.

G Comparing true survival curves with proposed method and the 2-phase semi-Markov model

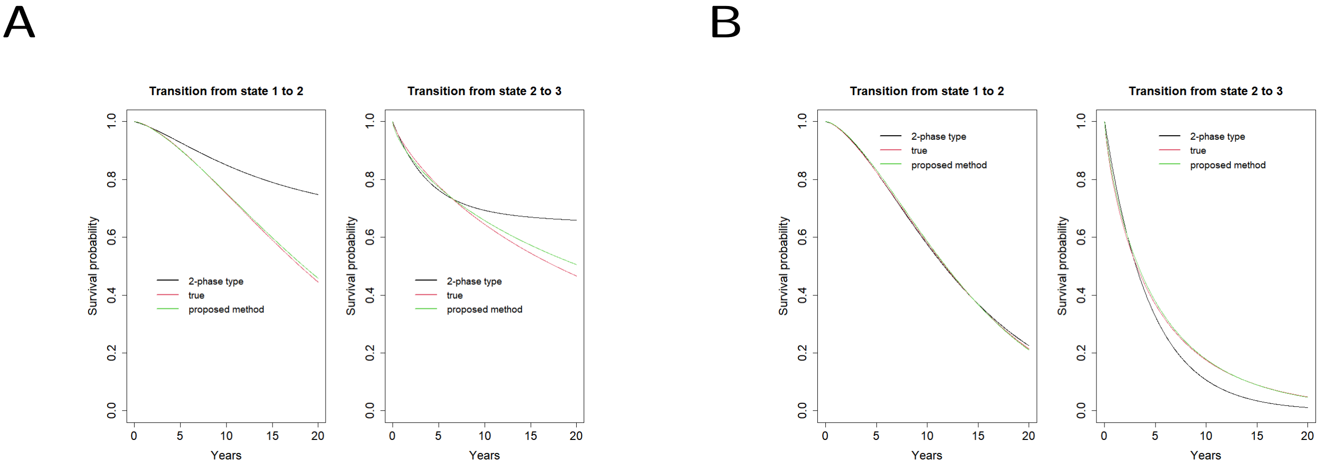

Figure S1 Comparing true survival curves (red line) for state 1 to 2 and state 2 to 3 with proposed method (green line) and the 2-phase type semi-Markov model by Titman and Sharples [7] (black line), implemented in **msm** package version 1.6.9 in **R**, using a Weibull + Weibull model specification without a covariate for $n = 5000$, point-wise averaged over 500 successful simulation runs. (**A**) Scenario I (55% AFs, 40% AAs, 5% CRCs) (**B**) Scenario II (30% AFs, 40% AAs, 30% CRCs).

H Results for the Norwegian data analysis

Table S2 Results of the Backward Stepwise Regression at $\alpha = 0.05$ Level of Significance of the Norwegian Data for Each of the Proposed Model Specifications with Covariates, Together with Results from Proposed Model Specifications without Covariates

Model	Parameter[a]	Estimate	P value	95% CI	AIC	BIC
M1	Exponential rate, λ_1	0.022	< 0.001	$(0.019, 0.025)$	2221.29	2230.85
	Exponential rate, λ_2	0.089	< 0.001	$(0.047, 0.132)$		
M2	Intercept, α_0	-4.381	< 0.001	$(-4.628, -4.133)$	2139.43	2158.54
	Age, α_1	0.027	< 0.001	$(0.013, 0.041)$		
	AT: AA, α_2	1.204	< 0.001	$(0.901, 1.507)$		
	Exponential rate, λ_2	0.089	< 0.001	$(0.047, 0.132)$		
M3	Exponential rate, λ_1	0.022	< 0.001	$(0.019, 0.025)$	2205.46	2224.57
	Intercept, β_0	-3.047	< 0.001	$(-3.878, -2.215)$		
	Age, β_1	0.085	< 0.001	$(0.030, 0.141)$		
	AT: AA, β_2	1.500	< 0.001	$(0.460, 2.539)$		
M4	Intercept, α_0	-4.378	< 0.001	$(-4.626, -4.131)$	2124.52	2153.19
	Age, α_1	0.027	< 0.001	$(0.013, 0.041)$		
	AT: AA, α_2	1.195	< 0.001	$(0.892, 1.498)$		
	Intercept, β_0	-3.033	< 0.001	$(-3.865, -2.200)$		
	Age, β_2	0.085	< 0.001	$(0.029, 0.140)$		
	AT: AA, β_3	1.456	0.01	$(0.414, 2.499)$		
M5	Exponential rate, λ_1	0.022	< 0.001	$(0.019, 0.025)$	2183.16	2197.49
	Weibull shape, $\log(\kappa_2)$	-1.938	0.01	$(-3.459, -0.417)$		
	Weibull scale, $\log(\theta_2)$	14.029	0.23	$(-8.814, 36.872)$		
M6	Intercept, α_0	-4.378	< 0.001	$(-4.625, -4.130)$	2101.52	2125.40
	Age, α_1	0.027	< 0.001	$(0.013, 0.041)$		
	AT: AA, α_2	1.201	< 0.001	$(0.898, 1.504)$		
	Weibull shape, $\log(\kappa_2)$	-1.921	0.01	$(-3.415, -0.426)$		
	Weibull scale, $\log(\theta_2)$	13.779	0.22	$(-8.317, 35.875)$		
M7	Exponential rate, λ_1	0.022	< 0.001	$(0.019, 0.025)$	2183.16	2197.49
	Weibull shape, $\log(\kappa_2)$	-1.938	0.01	$(-3.459, -0.417)$		
	Weibull scale, $\log(\theta_2)$	14.029	0.23	$(-8.814, 36.872)$		
M8	Intercept, α_0	-4.378	< 0.001	$(-4.625, -4.130)$	2101.52	2125.40
	Age, α_1	0.027	< 0.001	$(0.013, 0.041)$		
	AT: AA, α_2	1.201	< 0.001	$(0.898, 1.504)$		
	Weibull shape, $\log(\kappa_2)$	-1.921	0.01	$(-3.415, -0.426)$		
	Weibull scale, $\log(\theta_2)$	13.779	0.22	$(-8.317, 35.875)$		
M9	Weibull shape, $\log(\kappa_1)$	-1.685	< 0.001	$(-1.894, -1.477)$	1695.03	1709.36
	Weibull scale, $\log(\theta_1)$	9.844	< 0.001	$(8.058, 11.631)$		

(continued)

Table S2 (continued)

Model	Parameter[a]	Estimate	P value	95% CI	AIC	BIC
	Exponential rate, λ_2	0.058	< 0.001	$(0.032, 0.085)$		
M10	Weibull shape, $\log(\kappa_1)$	-1.638	< 0.001	$(-1.840, -1.435)$	1643.96	1663.07
	Intercept, α_0	12.487	< 0.001	$(10.077, 14.896)$		
	AT: AA, α_1	1.083	< 0.001	$(0.783, 1.383)$		
	Exponential rate, λ_2	0.059	< 0.001	$(0.032, 0.085)$		
M11	Weibull shape, $\log(\kappa_1)$	-1.680	< 0.001	$(-1.886, -1.474)$	1680.30	1704.19
	Weibull scale, $\log(\theta_1)$	9.805	< 0.001	$(8.052, 11.559)$		
	Intercept, β_0	-3.431	< 0.001	$(-4.210, -2.652)$		
	Age, β_2	0.081	< 0.001	$(0.028, 0.134)$		
	AT: AA, β_1	1.405	< 0.001	$(0.436, 2.374)$		
M12	Weibull shape, $\log(\kappa_1)$	-1.633	< 0.001	$(-1.834, -1.432)$	1629.51	1658.18
	Intercept, α_0	12.437	< 0.001	$(10.039, 14.836)$		
	AT: AA, α_1	1.081	< 0.001	$(0.775, 1.387)$		
	Intercept, β_0	-3.420	< 0.001	$(-4.288, -2.552)$		
	Age, β_2	0.081	< 0.001	$(0.025, 0.137)$		
	AT: AA, β_1	1.390	0.01	$(0.280, 2.499)$		
M13	Weibull shape, $\log(\kappa_1)$	-1.694	< 0.001	$(-1.902, -1.486)$	1652.67	1671.78
	Weibull scale, $\log(\theta_1)$	9.907	< 0.001	$(8.106, 11.709)$		
	Weibull shape, $\log(\kappa_2)$	-2.104	< 0.001	$(-2.971, -1.237)$		
	Weibull scale, $\log(\theta_2)$	17.186	0.03	$(1.916, 32.457)$		
M14	Weibull shape, $\log(\kappa_1)$	-1.646	< 0.001	$(-1.849, -1.442)$	1601.79	1625.68
	Intercept, α_0	12.561	< 0.001	$(10.117, 15.004)$		
	AT: AA, α_1	1.081	< 0.001	$(0.780, 1.382)$		
	Weibull shape, $\log(\kappa_2)$	-2.153	0.02	$(-3.935, -0.372)$		
	Weibull scale, $\log(\theta_2)$	18.087	0.29	$(-15.382, 51.555)$		
M15	Weibull shape, $\log(\kappa_1)$	-1.694	< 0.001	$(-1.902, -1.486)$	1652.67	1671.78
	Weibull scale, $\log(\theta_1)$	9.907	< 0.001	$(8.106, 11.709)$		
	Weibull shape, $\log(\kappa_2)$	-2.104	< 0.001	$(-2.971, -1.237)$		
	Weibull scale, $\log(\theta_2)$	17.186	0.03	$(1.916, 32.457)$		
M16	Weibull shape, $\log(\kappa_1)$	-1.646	< 0.001	$(-1.849, -1.442)$	1601.79	1625.68
	Intercept, α_0	12.561	< 0.001	$(10.117, 15.004)$		
	AT: AA, α_1	1.081	< 0.001	$(0.780, 1.382)$		
	Weibull shape, $\log(\kappa_2)$	-2.153	0.02	$(-3.935, -0.372)$		
	Weibull scale, $\log(\theta_2)$	18.087	0.29	$(-15.382, 51.555)$		

AIC, Akaike information criterion; BIC, Bayesian information criterion.

Note: Models M7, M8, M15, and M16 are the same as Models M5, M6, M13, and M14, respectively, after backward stepwise regression at $\alpha = 0.05$ level of significance.

[a] For transition times with Weibull specification, the parameter values were left in the log form because the delta method for approximating the CI resulted in a negative value for the lower bound of the CI. This is due to small sample size of the data.

References

1. Nelder JA, Mead R. A simplex method for function minimization. *Comput J.* 1965;7(4):308–313.

2. Broyden CG. The convergence of a class of double-rank minimization algorithms 1. General considerations. *IMA J Appl Math.* 1970;6(1):76–90.

3. Dubeau F. A remark on Richardson's extrapolation process and numerical differentiation formulae. *J Comput Phys: X.* 2019;2:100017.

4. Altman M, Gill J, McDonald MP. *Numerical issues in statistical computing for the social scientist.* vol. 508. John Wiley & Sons; 2004.

5. Jackson CH, et al. Multi-state models for panel data: the msm package for R. *J Stat Softw.* 2011;38(8):1–29.

6. Rice JA. *Mathematical statistics and data analysis.* Cengage Learning Inc; 2006.

7. Titman AC, Sharples LD. Semi-Markov models with phase-type sojourn distributions. *Biometrics.* 2010;66(3):742–752.
